# Nanomedicine strategies to improve therapeutic agents for the prevention and treatment of preterm birth and future directions

**DOI:** 10.1039/d2na00834c

**Published:** 2023-03-04

**Authors:** Jessica Taylor, Andrew Sharp, Steve P. Rannard, Sarah Arrowsmith, Tom O. McDonald

**Affiliations:** a Department of Chemistry, University of Liverpool Crown Street Liverpool L69 7ZD UK Thomas.Mcdonald@liverpool.ac.uk; b Harris-Wellbeing Preterm Birth Research Centre, Department of Women's and Children's Health, Liverpool Women's Hospital, University of Liverpool Crown Street Liverpool L8 7SS UK; c Department of Life Sciences, Manchester Metropolitan University Chester Street Manchester M1 5GD UK S.Arrowsmith@mmu.ac.uk; d Centre of Excellence in Long-acting Therapeutics (CELT), University of Liverpool Liverpool L7 3NY UK; e Department of Materials, Henry Royce Institute, The University of Manchester Manchester M13 9PL UK

## Abstract

The World Health Organisation (WHO) estimates 15 million babies worldwide are born preterm each year, with 1 million infant mortalities and long-term morbidity in survivors. Whilst the past 40 years have provided some understanding in the causes of preterm birth, along with development of a range of therapeutic options, notably prophylactic use of progesterone or uterine contraction suppressants (tocolytics), the number of preterm births continues to rise. Existing therapeutics used to control uterine contractions are restricted in their clinical use due to pharmacological drawbacks such as poor potency, transfer of drugs to the fetus across the placenta and maternal side effects from activity in other maternal systems. This review focuses on addressing the urgent need for the development of alternative therapeutic systems with improved efficacy and safety for the treatment of preterm birth. We discuss the application of nanomedicine as a viable opportunity to engineer pre-existing tocolytic agents and progestogens into nanoformulations, to improve their efficacy and address current drawbacks to their use. We review different nanomedicines including liposomes, lipid-based carriers, polymers and nanosuspensions highlighting where possible, where these technologies have already been exploited *e.g.* liposomes, and their significance in improving the properties of pre-existing therapeutic agents within the field of obstetrics. We also highlight where active pharmaceutical agents (APIs) with tocolytic properties have been used for other clinical indications and how these could inform the design of future therapeutics or be repurposed to diversify their application such as for use in preterm birth. Finally we outline and discuss the future challenges.

## Abstract 2

This review outlines the importance of improving the treatment of preterm birth, summarises the use of nanomedicines for the delivery of active pharmaceutical ingredients and discusses the opportunities/challenges for enhancing future patient outcomes.

## Graphical abstract

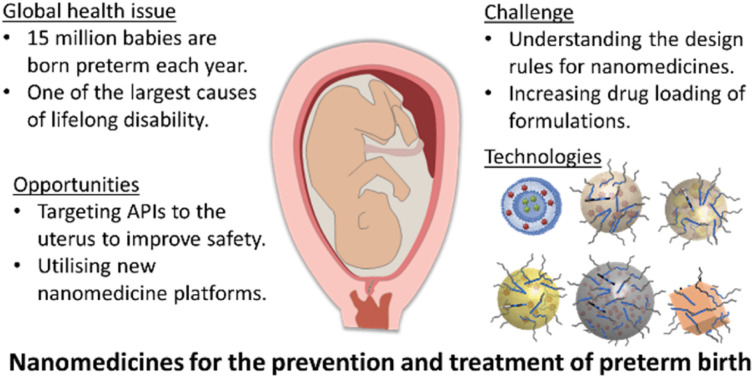

## Introduction

### Preterm birth

Preterm birth is defined as the birth of a child before 37 weeks' gestation; a full-term pregnancy lasts 39–41 week. According to the 2018 report from the World Health Organisation (WHO), preterm birth globally affects up to 15 million babies each year, with a total of 1 million infant mortalities.^1,2^ Globally the preterm birth rate is 11%,^3,4^ ranging from 8.7% in Europe to 13.4% in North Africa.^4^ Preterm birth is categorised according to gestation at delivery as; extremely preterm (<28 weeks), very preterm (28–32 weeks) and moderate to late preterm (32 to 37 weeks); with late preterm accounting for 70% of cases.^5^ Infants born at 22 weeks or with a birth weight of ≤500 g are considered to be at the lower limit of viability having very limited chances of survival.^6,7^ However, as gestational age at delivery increases, so does survival rate: Larroque *et al.* found neonates born at 24 weeks had an increased survival rate at 31%, followed by 78% at 28 weeks and 97% at 32 weeks.^8^ Survival rate however, is also dependent on the social and medical conditions in which the infant is born. Additionally, although the largest concern linked to preterm birth is immediate mortality, preterm infants often experience long-term morbidity including neurodevelopmental delay, cerebral palsy as well as respiratory disorders, cardiovascular disorders, infections, visual and hearing problems and learning disabilities owing to the underdeveloped fetal organs and systems.^9^ Indeed, preterm birth is one of the largest contributors to the disease burden and concomitant economic burden globally due to the lifelong disability often associated with being born preterm.^10^

### Clinical risk factors for preterm birth

Clinically, preterm birth is classified into different subtypes: In ∼30–35% of cases, preterm labour is pre-planned (medically indicated (iatrogenic)) and instigated by obstetricians; these planned cases occur primarily because the mother or the fetus is suffering from a potentially life-threatening condition and hence earlier delivery is safer.^11^ This includes poor fetal growth (fetal growth restriction, FGR) often as a result of a poor functioning placenta, fetal anatomical concerns, or fetal distress. Alternatively, planned preterm birth can occur if the mother has a health condition such as preeclampsia or other obstetrical complications including placental abruption. In these situations, obstetricians induce birth early given that there is a clear clinical advantage of early labour based on maternal and fetal considerations.^11,12^ Given that here, labour onset is pre-planned and somewhat controlled, pharmacological intervention is not necessary and hence iatrogenic preterm births are not the focus of this review.

The majority (∼65–70%) of preterm births are classed as sudden or unexpected and are referred to as spontaneous in onset.^11,13^ Spontaneous preterm birth results from either preterm premature rupture of membranes (PPROM) or idiopathic (no known cause) spontaneous labour onset involving uterine contractions and cervical dilation. Around 40–45% of all preterm births are idiopathic and are the main focus of the treatments described here.

Risk factors for spontaneous preterm birth include preterm birth in a previous pregnancy, having a short (≤25 mm) cervix (cervical insufficiency), either naturally or because of cervical surgery, and multiple pregnancy (*e.g.* twins). Other possible etiologies include intrauterine infection and inflammation. That previous spontaneous preterm delivery increases the risk of subsequent preterm birth, suggests that there may be a genetic factor involved. Many studies to date have identified multiple genetic variants linked to spontaneous preterm birth, however, in most cases, outcomes cannot be replicated across different population cohorts.^14^ Furthermore, the function of the genes identified are often not validated and therefore a biological role is not provided. In addition, many genetic studies to do not consider the gene–environment interactions including external and in utero environmental factors.

Multiple gestations, *i.e.* twins and triplets are considerably over-represented within preterm birth statistics accounting for 20% of all preterm births and 11.2% of all neonatal intensive care unit admissions despite representing only 3% of all live births. Up to 60% of multiple pregnancies will deliver <37 weeks' gestation and 10% <32 weeks. Nearly two thirds of multiple pregnancy preterm births are due to spontaneous onset of labour with the remaining third being iatrogenic due to maternal or fetal complications. Additionally, multiple gestations, account for more than 50% of preterm birth complications and 10–12% of all fetal deaths.^15–17^ Twins are at increased risk of low birth weight and as a result of preterm birth, have a five times higher risk of early neonatal and infant death than when only one baby is carried (singletons).^18,19^ The increased incidence of preterm birth in twins is multifactorial and includes maternal complications (discussed above) as well as uterine overdistention (stretch) which can trigger uterine contractions.

Interestingly maternal age, both ≤19 years and ≥35 years, is often described as high risk for preterm birth. For those of a young maternal age (≤19 years), spontaneous preterm birth may be due to biological immaturity increasing their risk of PPROM.^20^ Conversely recent studies suggest that advanced maternal age (≥35 years) and concomitant increase in risk of gestational diabetes and preeclampsia, may contribute to the increased risk of preterm birth.^21^ Pregnancies conceived by assisted reproductive technologies (*e.g. In vitro* fertilization/intracytoplasmic sperm injection, IVF/ICSI) are also at higher risk of preterm birth.^22^

### Prevention and treatment of preterm birth

The treatment of preterm birth can be divided into two categories; either prophylactic treatment for preterm birth prevention for women at high risk of preterm birth, such as those with a history of previous preterm birth and/or current evidence of a short cervix, or treatment at the onset of spontaneous preterm labour to suppress uterine contractions, known as tocolysis. The types of active pharmaceutical ingredients (APIs) used, and the clinical findings will be briefly discussed in this section.

**Fig. 1:**
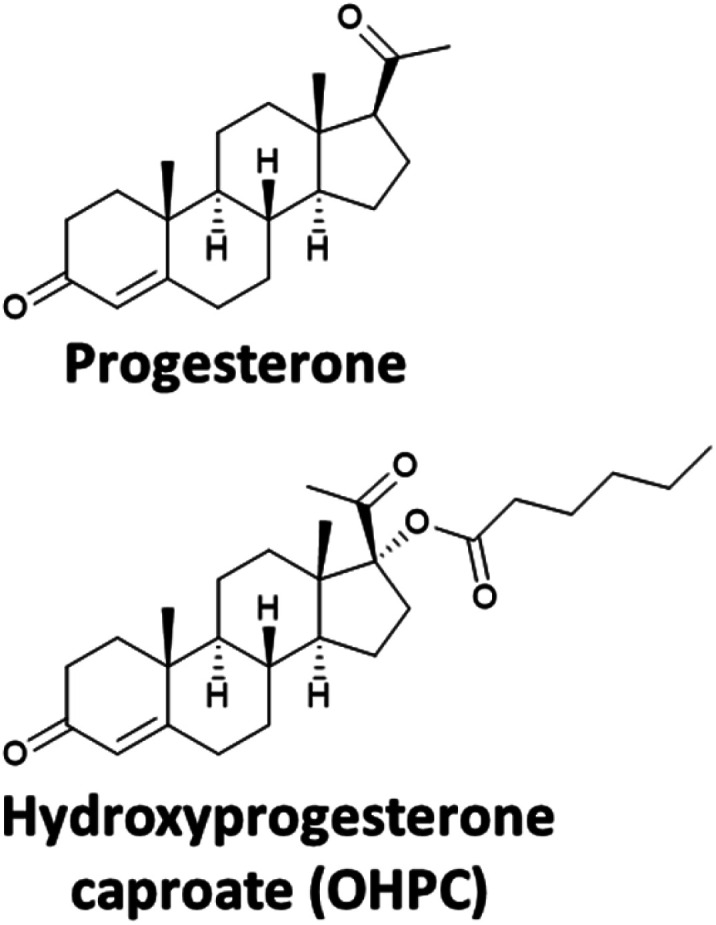
The structures, of progesterone and hydroprogesterone caproate (OHPC).

The APIs used for prophylactic treatment to reduce the risk of preterm birth occurring are progestogens (based on the hormone progesterone, Fig. 1) and include a synthetic derivative of progesterone, 17-hydroxyprogesterone caproate (OHPC) and vaginal progesterone. These two APIs have different physiochemical properties which influences their route of administration. For example, OHPC has a higher octanol–water partition coefficient (log *P*) and lower aqueous solubility than progesterone and is therefore administered as an intramuscular injection (Table 1). For women with a singleton gestation and a history of spontaneous preterm birth, OHPC (250 mg) can be administered weekly, starting at 16–20 gestational weeks until 36 weeks or delivery. For women with a short cervix, vaginal progesterone, either as a vaginal gel (90 mg) or micronised vaginal soft capsules (200 mg) is offered, however, the benefits of treatment are not clear: the largest study on vaginal administration of progesterone (200 mg capsules) (a multicentre, randomised and double-blind trial) reported that “Vaginal progesterone was not associated with reduced risk of preterm birth or composite neonatal adverse outcomes, and had no long-term benefit or harm on outcomes in children at 2 years of age.”^23^ However, a very recent network meta-analysis of clinical trials has shown that vaginal progesterone to be the treatment of choice for women with a singleton pregnancy and with a short cervical length.^24^ Indeed, in the United Kingdom, the National Institute for Health and Care Excellence (NICE) have recommended vaginal progesterone for high risk women. For OHPC, in 2003, a multi-centre, double-blind, placebo-controlled trial evaluating progesterone in women with prior history of spontaneous preterm birth found clear benefit with significant reduction in the rate of recurrent preterm birth and significantly prolonged the duration of pregnancy^25^ and was granted approval by the U.S. Food and Drug Administration (FDA). However, a subsequent follow-up confirmatory trial could not replicate the initial findings and raised other safety concerns.^26^ In October 2022, the FDA held a hearing to discuss the proposal to withdraw the approval of OHPC for the treatment of preterm birth, the decision has not yet been announced.

**Table 1:** The physiochemical parameters, dose and side effects of progesterone and hydroprogesterone caproate (OHPC) often used for prophylactic prevention of spontaneous preterm birth. Data on the side effects was taken from Dodd *et al.*^38^

API	log *P*	Solubility (mg ml^−1^)	Dose	Route of administration	Side effects
Progesterone	3.58	0.00546	200–400 mg	Oral, vaginal	- Maternal headache, breast tenderness, nausea, cough
Hydroxyprogesterone caproate (OHPC)	4.81	0.00086	250 mg	Intramuscular	- Maternal headache, breast tenderness, nausea, cough
					- Local irritation at injection site

For multiple gestations the aetiology of preterm birth is likely to be multifactorial and different to singleton pregnancies (for a review on this topic see Murray *et al.*^27^). For these pregnancies, there is currently a lack of evidence-based interventions for the prevention of preterm birth. The most studied is prophylactic progesterone administration (intramuscular OHPC and vaginal suppository or gel) in twin or triplet pregnancy. Neither vaginal progesterone in twin nor OHPC in twins or triplets has been shown to reduce preterm birth before 34 weeks.^28,29^ However, a higher dosage of daily vaginal progesterone (600 mg) for women with twins may improve preterm birth outcomes.^30^ Additionally, in a subgroup of women with twins and a short cervix, vaginal progesterone reduced the risk of delivering before 34 weeks.^31^ The same, however, has not been found for use of OHC in twin pregnancies and short cervix.^32^ Guidelines, such as those by the International Society of Ultrasound in Obstetrics and Gynecology (ISUOG), therefore recommend screening for preterm birth in twin pregnancy with cervical assessment on vaginal ultrasound but without clear guidance on management regime to follow. Due to insufficient evidence, routine use of intramuscular or vaginal progesterone for prevention of preterm birth in multiple pregnancies is not recommended.^33^ Unlike in singleton pregnancies, the use of tocolytics in multiple pregnancies has been less studied.^34^ Questions also arise over whether tocolytic treatments demonstrate the same efficacy in preventing or delaying preterm birth or improving infant outcomes for multiple gestation pregnancies. There is insufficient evidence for the prophylactic use of β2 adrenergic receptor agonists in twins,^35^ whilst nifedipine appears safe and effective in delaying labour in both singleton and twin pregnancies with spontaneous preterm labour onset.^36^ Indomethacin has not been investigated specifically in multiple pregnancy, but twin gestations were included in a network meta-analysis comparing tocolytic therapies for preterm delivery. Indomethacin showed the highest delay in delivery by 48 hours and least maternal side effects,^37^ albeit its use was restricted to before 32 weeks due to known fetal complications. Given the weaker evidence basis for the preterm birth treatments for multiple gestations compared to singleton pregnancies more research is needed to identify the best treatment options.

**Fig. 2:**
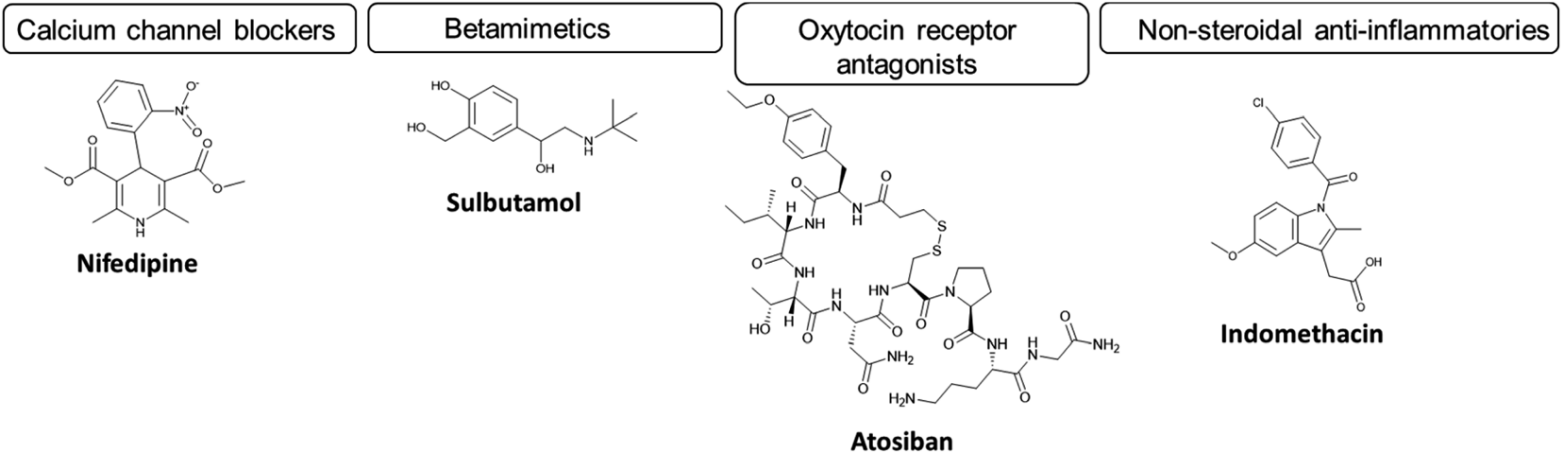
The main subcategories of commonly used tocolytics and their structures.

In cases of spontaneous labour onset with uterine contraction and/or cervical dilation, women are primarily treated with APIs named tocolytics, uterine contraction suppressants. The primary role of tocolytics is to reduce the frequency and intensity of uterine contractions.^39–41^ The resulting delay in labour is usually sufficient to enable the transfer of the woman to a care setting with appropriate neonatal facilities, or for administration of corticosteroids (betamethasone or dexamethasone) to enhance fetal lung maturation prior to delivery; to reduce neonatal respiratory complications. Furthermore, the woman may also be administered magnesium sulphate to provide neuroprotection and reduce brain injury in the infant. These APIs can be divided into subcategories of tocolytics based on their mechanism of action (Fig. 2). Tocolytics include calcium channel blockers (*e.g.* nifedipine), β2 adrenoceptor agonists (betamimetics) (*e.g.* salbutamol, ritodrine, terbutaline), oxytocin receptor antagonists (*e.g.* atosiban, nolasiban and barusiban) and non-steroidal anti-inflammatory drugs (NSAIDs) (*e.g.* indomethacin).^37,42,43^ For further discussion of mechanism of action of these drugs, see Arrowsmith *et al.* 2010 and Wray *et al.* 2022.^44,45^ These tocolytic APIs, with the exception of salbutamol, generally possess limited aqueous solubility (with predicted values ≤0.05 mg ml^−1^), while all except atosiban can be administered orally (Table 2). Each API presents side effects to the mother and/or the fetus which can be seen in Table 2. It is important to note however, that very few tocolytics are approved for the treatment of preterm birth. Hence, given the very limited range of clinical options, and the limited evidence of the benefit of the approved treatments, most are used off-label for the treatment of preterm birth. The only approved treatment is atosiban, which was approved by the European Medicines Agency (EMA) in 2000, but not the FDA. Atosiban is an oxytocin-receptor antagonist (ORAs) developed especially for the treatment of preterm birth. Because the primary targets for oxytocin are the uterine muscle (myometrium) and decidua, it was anticipated that atosiban would be highly organ specific with limited adverse side effects. Atosiban displayed mixed results in clinical trials; it has been shown to reduce the likelihood of birth within the first 48 hours,^46^ however without improvement in neonatal outcomes. An upcoming multi-centre trial of atosiban and steroid *vs.* placebo has been approved which may address the neonatal benefit question.^47^ Several other ORAs are also in development including barusiban and nolasiban which confer different pharmacokinetic profiles and potencies.

**Table 2:** Commonly used tocolytics showing their physiochemical parameters, dose and side effects. The data on side effects of the drugs was obtained from the following ref. ^48–50^ The calculated log *P* values and calculated solubilities were ALOGPS values^51^

API	log *P*	Solubility (mg ml^−1^)	Dose	Route of administration	Side effects
Nifedipine	2.49	0.0177	10–30 mg, then 10–20 mg every 4–6 h	Oral	- Maternal headache and hypotension
					- Tachycardia (maternal and fetal)
Sulbutamol	0.44	2.15	10 mcgs min^−1^, titrated to response until contractions cease (max rate 45 mcgs/min), then gradually reduce, max duration 48 h	Oral or IV	- Maternal tachycardia, hyperglycaemia and pulmonary tachycardia
					- Fetal tachycardia and respiratory distress syndrome
Atosiban	−0.17	0.0517	6.75 mg bolus, then 300 mg min^−1^ infusion for 3 h, followed by 100 mg min^−1^ infusion for up to 45 h	IV	- Maternal tachycardia and chest pain
Indomethacin	4.25	0.0024	100 mg, then 25 mg orally 4–6 hourly for 48 h	Oral	- Fetal reduced amniotic fluid, premature closure of ductus arteriosus

Despite there being several different tocolytics available, they have a number of clinical drawbacks. Firstly, tocolytics have repetitive dosing and time restrictions; with many requiring the termination of administration after 48 hours.^52^ Secondly, their efficacy is variable amongst different patient cohorts with some displaying uterine quiescence for up to 7 days, whereas others only maintain quiescence for 24 hours after the maximum dosing of 48 hours.^53^ Thirdly, further speculation around the safety and efficacy of tocolytics is raised due to a number of associated side effects in the mother and/or the fetus, the nature of which depend on the subclass of tocolytic used (see Fig. 2).

Currently in clinic, calcium channel blockers are the most common first line treatment due to their enhanced tocolytic activity over other tocolytic categories and reduced fetal effects.^54,55^ However, owing to the fact that the same calcium channels are present in the heart and cardiovascular system, calcium channel blockers can cause substantial maternal side effects including hypotension and tachycardia.^50^ Hence, calcium channel blockers suffer from time restrictions of use up to 48 hours and dosage restrictions to a maximum of 160 mg day^−1^.^56^ Indomethacin is another commonly used tocolytic. It reduces the activity of the type 2 isoform of cyclo-oxygenase (COX-2), which is important for the production of prostaglandins within intrauterine tissues, and has been shown to reduce uterine contractility; thus, extending gestation. However, it also poses concerning side effects including premature closure of a major blood vessel, the ductus arteriosus, in the fetus as well as intraventricular bleeding.^57,58^

With significant disadvantages still associated with the most potent tocolytics, there is a clear requirement for the development of new tocolytics or new formulations of pre-existing APIs that can be engineered to improve their pharmacokinetic and safety properties. The key desirable outcomes are to improve the *in vivo* pharmacological performance of tocolytic agents, whilst addressing dosage restrictions and maternal and fetal safety concerns. The development of new APIs for the treatment of preterm birth is outside the scope of this review, instead the reader is directed to the review by Zierden *et al.*^59^

### Potential advantages of using nanomedicine for preterm birth

Nanomedicine, the application of nanotechnology for therapeutics and diagnostics, has provided significant advantages for medicine in recent decades.^60–62^ Most notably, nanomedicine technologies offer the potential to alter the pharmacokinetics and biodistribution of APIs. An example of this is in the clinical success of targeted liposomes for the delivery of anticancer therapies: paclitaxel loaded liposomes for lung cancer, using d-α-tocopheryl polyethylene glycol 1000 succinate triphenylphosphine conjugate to actively target the mitochondria in cancerous cells, have shown a strong inhibitory effect of 73% and increased apoptosis of cancerous cells.^63^ In the setting of preterm birth, better drug pharmacokinetics and biodistribution to reduce the dosage profile is clearly attractive for improving the clinical performance of tocolytic agents where side-effects in both the mother and the fetus are often limiting factors for their use. As noted for the example in cancer therapy, the targeting of nanomedicines can be used to further enhance the pharmacokinetics of an API, typically using intravenous (IV) as the route of administration. Nanomedicines can be designed to incorporate passive and/or active targeting. Passive targeting uses the properties of the nanoparticles themselves (diameter, surface charge, surface coating) in combination with the physiology of the disease site to increase the percentage of the API that reaches the target site. An example of this such of targeting is the enhanced permeation and retention effect which exploits the pathophysiological characteristics observed in some solid tumours.^64,65^ Active targeting involves the conjugation of a high-affinity ligand onto the surface of the nanoparticle. This ligand is then able to bind to a specific receptor that is found on the targeted cells.^66^ Targeting of nanomedicines is a considerable opportunity for improving the efficacy of treatments.

Another opportunity offered by nanomedicines is the potential to open new routes of administration. For ‘conventional’ (non-nano) formulations of an API the route of administration is limited by the physicochemical parameters of the API. For example, for oral dosing the API needs to typically possess physiochemical parameters within Lipinski's rule of 5 (molecular weight ≤500, log *P* ≤ 5, H-bond donors ≤5, H- bond acceptors ≤10).^67^ This is because the oral bioavailability is controlled by the aqueous solubility and permeability of the drug through the gastrointestinal membrane; APIs with low solubility and low permeability are often associated with poor therapeutic efficacy after oral administration.^68^ However, the use of nanomedicines can be used to circumvent the limits caused by an API's physicochemical properties. For example, nanosuspensions can be used to enhance the dissolution kinetics of poorly solubility APIs,^69^ while lipid nanoparticles can show mucoadhesive behaviour in the gut or drive lymphatic uptake of their payload.^70^ Such approaches are of interest for a wide range of conditions including cancer.^71^ Conversely, moving from an oral dose to an IV nanomedicine can be beneficial, as the properties of the nanomedicine can influence the elimination mechanisms. Thus the properties of the nanomedicine will also influence the pharmacokinetics of the treatment rather than these being purely controlled by the physiochemical properties of the dissolved API. In the treatment of preterm birth, where therapies are given in a clinical setting (compatible with IV administration), it may be attractive to move from the oral dosing of an API to IV dosing of a nanomedicine with the aim of improving targeting to the uterus. This could potentially allow for a dose reduction and reduced side-effects. Furthermore, because nanomedicines can offer different behaviour in terms of penetration across biological barriers there is also the potential to delivery APIs topically or vaginally. Vaginal administration is an attractive route for the treatment of preterm birth as it avoids hepatic first-pass metabolism that is seen for oral dosing and is also successful in delivering a local effect at the uterus.^72,73^ The vagina is lined with mucosal tissue and as such the efficacy of vaginally administered APIs is strongly influenced by the interaction of the formulation with the mucus lining in the vagina. In terms of nanomedicine, both mucoadhesive or mucoinert nanoparticles can be designed, each with its own strengths and weaknesses. Mucoadhesion can be used to prolong the residence time of an API in the vagina, however, this can also limit the penetration of the API through the mucus to the underlying cells.^74^ Conversely mucoinert/mucus penetrating nanoparticles can improve the delivery of an API to the vaginal epithelium.^75^ The ability to introduce specific moieties to target specific organs and the ability to address the aqueous solubility issues surrounding the lipophilic character of many drugs, including tocolytics, makes this technology exciting and viable for novel therapies for preterm birth, as discussed next.^76^

### Nanomedicine opportunities for tocolytics

**Fig. 3:**
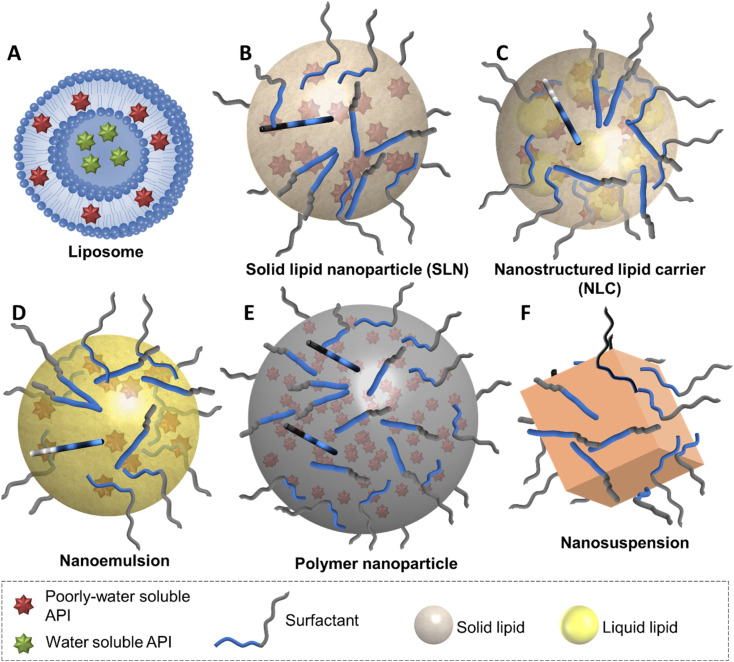
Illustration of liposomes, solid lipid nanoparticles, nanostructured lipid carriers, nanoemulsions, polymer nanoparticles and nanosuspension nanomedicines showing the differences in the structure and composition of the different nanoparticles.

The opportunities for tocolytic nanomedicine formulations are vast, particularly due to the hydrophobic nature of the majority of the tocolytic APIs. Some of the nanomedicine types that are best suited to the delivery of poorly water-soluble APIs are liposomes, solid lipid nanoparticles, nanostructured lipid carriers, nanoemulsions, polymer nanoparticles and nanosuspensions (Fig. 3).

Whilst beneficial, the ability of nanomedicines to alter the pharmacokinetics and biodistribution of an API also introduces additional complexity to the formulation. A nanomedicine has many additional physical properties when compared to a dissolved API including the size, shape, surface coating and particle composition, which may all influence the biological behaviour of the nanomedicine.^62,77,78^ Additionally, these particle properties can typically interact with one another and so it can be challenging to tailor the nanomedicine properties to a given application. Furthermore, the efficacy of a nanomedicine is strongly influenced by its behaviour *in vivo*, including drug release from the nanoparticles, nanomedicine accumulation in the target cells (*e.g.* the uterus in preterm birth) and its biodistribution. Typically, initial development of nanomedicine formulations focusses on producing stable nanoparticles with the required surface coating, the highest possible drug loadings (measured as a percentage of the drug in the total formulation) and the appropriate drug release kinetics. The ability to obtain the highest drug loadings possible for a formulation is important for the potential translation of a nanomedicine formulation to clinical use. While nanomedicines offer the potential to increase the therapeutic index of an API,^79^ allowing for dose reduction. Nanomedicine formulations also can consist of a considerable mass of excipients which reduce the drug loading in the formulation; ultimately low drug loadings can make it impractical to dose the therapeutically relevant amount of the API. Nanoparticle diameter is another key property and is possibly the most studied parameter with numerous studies investigating how this influences the biological behaviour of nanoparticles. The diameters of the nanoparticles produced are often driven by what is possible due to the processing method and nanomedicine type used; typically, it is possible to produce samples with diameters that vary up to five-fold. While the size of a nanomedicine is not the only factor that controls its biological properties, some generalisations have been provided. For IV dosing, a the distribution of particle sizes can not exceed 5 μm in diameter, this is because particles larger than this may lead to embolization.^80^ Nanoparticles in the diameter range of 20–200 nm do not generally leave the capillaries, while nanoparticles with diameters ≤10 nm can be filtered out of the circulation by the kidneys.^81^ For oral administration, there is no upper particle size limit and generally smaller sizes of nanoparticles can increase bioavailability of the API.^82^ The understanding of the effect of nanoparticle size on less common administration routes (such as vaginal pessaries) is more limited; we will discuss the findings of specific studies in the later sections of the review.

In the treatment of preterm birth with tocolytics, the ultimate objective is to target the distribution of the API to the uterus to stop contractions and avoid effects in other organs and systems in the mother, as well as critically, to prevent the API from crossing the placenta and entering the fetal circulation. In terms of applications in the treatment of preterm birth, there are very few studies that have systematically varied particle size and investigated biodistribution. One study has shown that smaller gold nanoparticles (20 or 50 nm) displayed higher accumulation in the uterus than nanoparticles with diameters of either 100 or 200 nm.^83^ However, this behaviour might not be generalisable to other nanoparticles with different surface properties. In the following sections we review the different nanomedicine systems and discuss how they have been developed and investigated to date for the treatment of preterm birth, including the *in vitro* and *in vivo* models used to evaluate their efficacy. We also discuss nanomedicine formulations that have been developed for indications other than preterm birth but with APIs that have known tocolytic properties and could therefore be repurposed for use in treating preterm birth. To aid comparison of the different nanomedicine formulations each section contains a table that summarises the key feature of the formulations.

### Liposomes

**Fig. 4:**
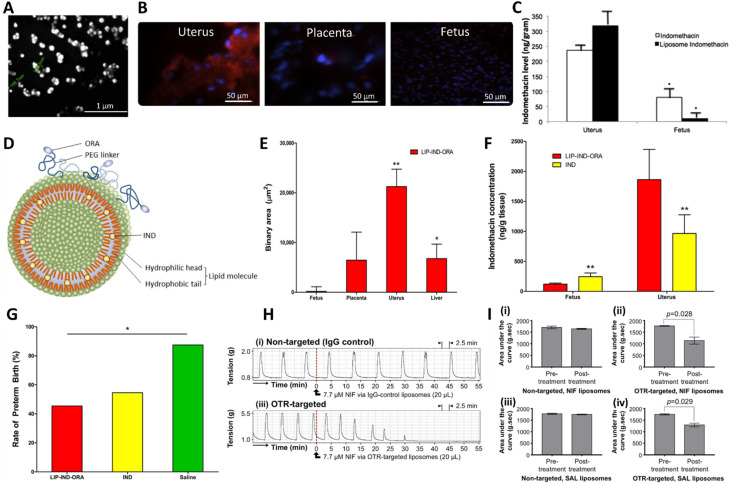
Liposomes developed for the treatment of preterm birth. (A) Scanning electron microscopy of indomethacin loaded liposome. (B) Analysis of the distribution of fluorescently labelled (red) indomethacin loaded liposomes in uterus, placenta, and fetus. (C) Quantification of the indomethacin concentration within uterus and fetus 4 hours following administration of indomethacin or indomethacin loaded liposomes. (D) Illustration of indomethacin (IND) loaded liposomes with oxytocin receptor antagonist (ORA) targetting ligands. (E) Biodistribution of liposomes containing indomethacin and targeted with oxytocin receptor antagonist components (LIP-IND-ORA) *in vivo* in pregnant mice. (F) A comparison of the targeted indomethacin liposomes (LIP-IND-ORA) and free indomethacin (IND) in terms of the concentrations of indomethacin in the maternal uterus and fetus, both were dosed at the same concentration (1 mg kg^−1^). (G) Comparison of the *in vivo* therapeutic efficacy in prevention of LPS induced preterm birth, free indomethacin and indomethacin liposomes were both dosed at 1 mg kg^−1^. (H) Effect of non-targeted and OTR-targeted nifedipine loaded liposomes on myometrial contractions *in vitro*. (I) Analysis of the contractility testing to obtain area under the curve (AUC) values for 30 minutes immediately prior to and 30 minutes after treatment with nifedipine (NIF) loaded liposomes (pre-treatment and post-treatment, respectively), (i) non-targeted liposomes containing NIF, (ii) targeted liposomes containing NIF, (iii) non-targeted liposomes containing salbutamol (SAL) and (iv) targeted liposomes containing SAL. (A), (B) and (C) are adapted with permission from Elsevier reference,^85^ (D), (E), (F) and (G) are taken from reference under Creative Commons CC BY license,^76^ (H) and (I) are taken with permission from Elsevier ref. 86.

Liposomes are artificial spherical vesicles made of one or more lipid bilayers.^84^ Due to this structure, a liposome possesses an aqueous core that is surrounded by a hydrophobic lipid bilayer(s). As such, liposomes can encapsulate both hydrophilic drugs (in the aqueous core) and hydrophobic drugs (within the lipid bilayers(s)) (Fig. 3A). Liposomes are the nanomedicine technology platform that has been investigated most extensively for the delivery of tocolytics. To the best of our knowledge, Refuerzo *et al.* were the first to develop liposomal formulations containing indomethacin, for the prevention of preterm birth.^85^ Indomethacin is an attractive tocolytic to formulate into the lipid bilayers of liposomes due its lipophilic nature (log *P* of 4.5). Refuerzo *et al.* developed fluorescently labelled multilamellar liposomes (150–170 nm) (Fig. 4A) synthesised from phosphatidylcholine and cholesterol *via* the lipid hydration-extrusion technique and the liposomes contained 3.7 wt% indomethacin.^85^ Fluorescently-labelled particles were imaged to identify particle accumulation, which showed successful, predominantly uterine accumulation (Fig. 4B) and minimal evidence of placental transfer (Fig. 4C).^85^ The liposomes were further developed to specifically target oxytocin receptors (OTRs) in the uterus, where they are abundantly expressed and are responsible for mediating the contraction stimulating effect of oxytocin during labour.^76^ Refuerzo *et al.* targeted the liposomes to the OTRs, by conjugating atosiban, a competitive OTR antagonist, to the surface of the liposome (Fig. 4D).^76^ Analysis of these OTR-targeted liposomes in pregnant mice *via* tail vein injection showed predominant distribution in the uterus compared to other sites such as the liver, placenta and fetus (Fig. 4E). When comparing the indomethacin-loaded, targeted liposomes to free indomethacin, the targeted liposomes demonstrated a 4-fold reduction in placental indomethacin concentration and hence reduced placental transfer (Fig. 4F). Finally, in an infection-induced preterm birth mouse model using lipopolysaccharide (LPS), the targeted liposomes reduced preterm birth rates compared to control (Fig. 4G).^76^ Further to these studies, Paul *et al.* developed uterine-targeted liposomes of ∼200 nm diameter containing other tocolytic agents: nifedipine, salbutamol, rolipram and indomethacin.^86^ The liposomes contained excipients cholesterol and the phospholipid 1,2-distearoyl-*sn*-glycero-2-phosphocholine (DSPC), as adapted from a method developed by Hua *et al.* for the development of antibody-conjugated liposomes.^87^ To add a uterine targeting functionality, a lipid 1,2-diastearoyl-*sn*-glycero-3-phospho-ethanolamine coupled to polyethylene glycol (PEG) with a maleimide chain end functionality was conjugated to an anti-OTR antibody. The liposomes were then synthesised by high pressure extrusion.^86^ Their key findings showed that OTR-targeted liposomes containing nifedipine, salbutamol or rolipram were able inhibit *ex vivo* human uterine contractions, whilst non-targeted liposomes did not reduce contractions at the equivalent concentrations tested (Fig. 4H).^86^ A reduction in contractility was only observed when the targeting approach was used (Fig. 4I). In a similar inflammation-induced mouse model of preterm birth, indomethacin-loaded OTR-targeted liposomes reduced preterm birth rates to 18%, whereas non-targeted liposomes had no significant effect and remained at a preterm birth rate of 58%.^86^ Further work published by Hua *et al.* exploring the *in vitro* mechanisms of the cellular uptake, internalisation and toxicity profiles of liposomes with a mean diameter of 200 nm.^88^ While the encapsulation efficiency was reported in the work, the drug loadings in the liposomes were not given. They showed that the incorporation of OTR targeting enhanced cellular interactions with uterine tissues, with the potential to increase tocolytic efficacy and decrease dosage profiles.^76,86,89^ This was true regardless of the targeting ligand (either an OTR monoclonal antibody or the OTR antagonist, atosiban).^88^ Both targeting approaches enhanced cellular internalisation compared to conventional liposomes; +78% with OTR antibody targeting and +82% with atosiban targeting.^88^

From the targeted liposomal studies, it is evident that the conjugating of a targeting moiety such as one directed to the OTR and exploiting the upregulation of OTRs in pregnant uterine tissue is a significant angle to explore for the progression of tocolytic agent development. Nevertheless, although the OTR has shown significant targeting benefit, other receptors that are over expressed and/or are uterine- or gestationally-specific should also be considered as future therapeutic targets.^90^ However, to date, other than the family of prostaglandin receptors which also change expression with gestation^91^ there have been very few alternatives put forward.

While liposomes have shown exciting data in terms of enhancing the efficacy of tocolytic APIs, further work is required to progress these systems through the preclinical translation pathway. Additionally, liposomes typically show limited drug loading, *e.g.* for indomethacin the highest drug loading reported is only 3.7% (a summary of all the liposomal systems is given in Table 3).^76^ Furthermore, long term storage instability in the dispersed form can present another potential barrier to the clinical use of liposomes.^92^ Therefore, alternative nanomedicine platforms may help to address these challenging areas by providing increased drug loading and greater storage stability.

**Table 3:** Liposomes containing APIs with tocolytic properties^a^

API	Lipid(s)	Targeting	Mean diameter (nm)	Drug loading (wt%)	Administration route	Ref.
Indomethacin	Soy bean phosphatidyl- choline & cholesterol	N/A	150–170	3.7	IV	85
Indomethacin	Soy bean phosphatidyl- choline, cholesterol & 1,2-distearoyl-*sn*-glycero-3-phosphoethanolamine (DSPE)	Atosiban-PEG_2000_-DSPE	124	3.7	IV	76
Nifedipine, salbutamol, rolipram or indomethacin	1,2-Distearoyl-*sn*-glycero- 2-phosphocholine (DSPC), cholesterol & DSPE	Polyclonal anti-OTR antibody-PEG_2000_-DSPE	∼200	*	IV	86
Nifedipine or salbutamol hemisulfate	DSPC, cholesterol & DSPE	Polyclonal anti-OTR antibody-PEG_2000_-DSPE	200	*	Not tested *in vivo* (however route of administration would be IV)	58
		Atosiban-PEG_2000_-DSPE				

a * values were not reported in the paper and could not be calculated based on the information given in the experimental.

### Lipid-based nanomedicines

Lipid-based nanomedicines are nanoscale particles that are made of a lipid core. An attractive feature of these nanomedicines is that the lipid core of the particle can be made from physiological or biodegradable lipids.^93^ As with liposomes, the lipids within the particles can then processed within existing biological pathways within the body. Lipid-based nanomedicines can be categorised based on their core composition; solid lipid nanoparticles (SLNs) are made of lipid(s) that are solid at body temperature (Fig. 3B), nanostructured lipid nanocarriers (NLCs) have a core that contains both solid and liquid lipids (Fig. 3C) and nanoemulsions have a lipid core that is liquid (Fig. 3D). In all cases, the lipid-based nanomedicines have a coating of a stabiliser that provides colloidal stability.

To our knowledge, there are no reported examples of any of these three lipid-based nanomedicines having been used to trial the delivery of tocolytics for the treatment of preterm birth in any *in vitro*, *ex vivo* or *in vivo* model. However, lipid-based nanomedicines containing APIs with tocolytic properties have been produced but for different indications. Formulations of progesterone have also been developed with potential prophylactic uses.

SLNs were introduced as a new potential nanocarrier system in 1991.^94^ They sparked further interest within the nanomedicine field following a publication by Muller *et al.* in 2000, that successfully highlighted the opportunities amongst SLN systems for pharmaceutical translation.^94^ SLNs were developed to combine the advantages of liposomes such as high chemical and physical stability, biocompatibility, specific targeting capabilities and sustained release.^95–97^ Moreover, their composition of physiologically-compatible excipients affords other pharmacological benefits such as improvement of bioavailability and decreased toxicity, with a simultaneous increase in efficacy for comparable dosages to non-formulated APIs.^98,99^ The ability for lipid-based nanoparticles to encapsulate an API is strongly influenced by the log *P* of the API and its compatibility with the lipids used in the formulation. Typically, high log *P* APIs are more successfully incorporated into lipid nanoparticles.^100,101^ Additionally, the surfactants selected in formulation, play a significant role in the formation of colloidally-stable nanoparticles. Recently, it has also been found that subtle differences in the relative block lengths of polymer surfactants can influence the polarity inside a lipid nanoparticle.^102^ Therefore, the insights offered by successful formulations reported in the literature could potentially be used to inform the design of future formulations.

**Fig. 5:**
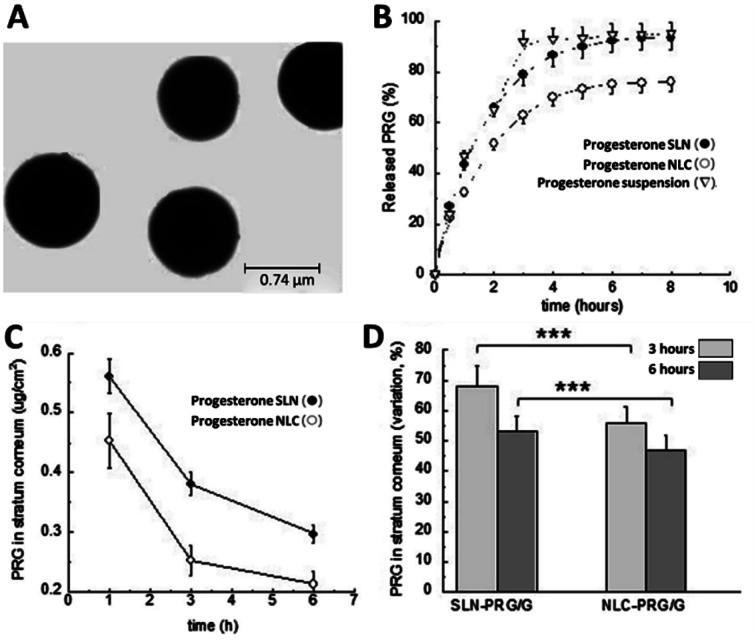
Lipid-based nanomedicines containing APIs relevant to the treatment of preterm birth. (A) TEM analysis of solid lipid nanoparticles based on l-cysteine. (B) *In vitro* release kinetics of progesterone from SLNs, NLCs compared to the suspension of progesterone in poloxamer 188 (2.5% w/w). (C) Analysis of the amount of progesterone in the stratum corneum after the application of either the SLNs or NLCs. (D) Comparison of the progesterone concentration in the skin after treatment with either the SLNs or NLCs after 3 hours or 6 hours in stratum corneum, as determined by tape-stripping experiments. (A) was adapted from reference under Creative Commons license,^109^ (B), (C) and (D) were adapted with permission from Elsevier ref. ^111^.

SLNs have been successful at encapsulating a range of therapeutic agents for several acute and chronic conditions including ocular diseases and anticancer therapeutics.^103,104^ Of particular interest to the treatment of preterm birth, is lipid-based nanoformulations of indomethacin which were developed originally for ocular inflammation and rheumatoid arthritis due to its anti-inflammatory properties.^103,105^ For the treatment of chronic ocular inflammation, Hippalgaonkar *et al.* synthesised indomethacin loaded SLNs *via* a hot homogenisation technique with Compritol 888 ATO as the lipid to produce 1.9 wt% indomethacin nanoparticles with a mean diameter of 140 nm. They showed that the mean particle size of the SLNs could be tuned by varying the composition of the Tween 80 and poloxamer 188 surfactant mixture used. The SLN formulation was compared directly against Indocollyre^®^- a commercially available indomethacin eye drop containing 0.1 wt% of drug; showing an increased corneal permeation without effecting corneal integrity, and thus suggested improved safety and efficacy for the treatment of ocular inflammation.^103^ Balguri *et al.* then published using indomethacin SLNs to the deliver the API to posterior segment ocular tissues for the treatment of conditions such as macular oedema and ocular inflammation. They used the same excipients as Hippalgaonkar *et al.* but also functionalised the nanoparticles with chitosan chloride to enhance ocular tissue penetration. Chitosan is a linear polysaccharide derived from the chitin shells of crustaceans with adhesive-like properties and has been shown to increase mucoadhesion and potentially increase nanoparticle transport across the epithelium.^106^ The chitosan functionalised SLNs had a mean diameter of 265 nm and were found to increase the ocular penetration of indomethacin across the cornea compared to non-functionalised SLNs.^107^ Another API with tocolytic properties that has been formulated as an SLN is nifedipine. Barman *et al.* made particles of hydrogenated soybean phosphatidylcholine and dipalmitoylphosphatidyl glycerol with a mean diameter of 69 nm and a drug loading of nifedipine of 2.7%. By using trehalose as a cryoprotectant, the SLNs could be freeze dried to give a re-dispersible formulation which was stable under storage in the dry form for at least 6 months.^108^ Cassano and Trombino also processed progesterone to produce SLNs with the aim to develop a formulation with sustained release.^109^ They functionalised the SLNs with l-cysteine amino acid units designed to increase the time enabled for SLNs to anchor into the mucus layer of the uterus. The particles were produced by a microemulsion method, and the unloaded nanoparticles had a mean diameter of 740 nm (Fig. 5A) (the progesterone loaded particles were slightly smaller with a mean diameter of 640 nm). The drug loading was not specifically reported but we calculated it to be 3%. This study concluded that prolonged progesterone release was achieved for up to 72 hours in acidic *in vitro* media (representative of the vaginal cavity pH), therefore highlighting that progesterone-SLNs may be a viable route to provide sustained intravaginal progesterone administration, such as that which is required during assisted reproduction treatment *e.g.* for luteal support during embryo transfer and implantation.^109^ Importantly however, these studies also demonstrate the potential for SLNs to act as viable drug delivery vehicles for APIs for the treatment of preterm birth.

Nanostructured Lipid Carriers (NLCs) were developed as ‘second generation lipid nanoparticles’ to overcome drawbacks associated with some SLN formulations^118,119^ including potential for low drug loading and drug expulsion resultant of the highly crystalline nature of the solid lipid cores.^119,120^ As a result, NLC formulations are very closely linked to SLNs in terms of their composition, however with an additional liquid lipid in the core of the carrier (Fig. 3), NLCs tend to display higher drug loading capabilities. NLCs of indomethacin have been produced by modifying an SLN formation with the addition of a liquid lipid, Miglyol 812, to the solid lipid of Compritol 888 ATO. The resulting nanoparticles had a mean diameter of 227 nm and a drug loading of 9.9%.^107^ This drug loading was much higher than that of the SLNs with a similar composition (excluding the liquid lipid) which had a 1.9% drug loading. *In vivo* application of the NLC formulation produced a 5-fold higher concentration of indomethacin to ocular tissues than the SLN formulation, which was attributed to the higher drug loading.^107^ NLCs of indomethacin and a non-tocolytic API (celastrol) have also been produced for potential applications in the treatment of rheumatoid arthritis as a transdermal drug delivery system. The optimised formulation contained as Precirol ATO 5 (solid lipid) and Labrasol ALF (lipid) and had a mean diameter of 27 nm with a total drug loading of 3.6%. The particles showed no irritation on rat skin but successfully penetrated through the top ∼300 μm of skin, demonstrating the potential of these lipid carriers to enhance API penetration into epithelial tissue.^105^

There are a few examples of NLCs of progesterone, although none specifically focussed on preterm birth treatment. Yuan *et al.* in 2007 produced NLCs by melt-emulsification using monostearin and stearic acid as the solid lipids, with oleic acid as the liquid lipid. Mean particle sizes of 340–385 nm were obtained (reported as a volume average rather than intensity average as is typical for dynamic light scattering (DLS)) and drug loadings were in the range of 3–11%.^110^ In 2017, Esposito *et al.* published work comparing both SLN and NLC formulations of progesterone using both ultrasound homogenisation and high-pressure homogenisation. The high-pressure homogenisation approach avoided the production of agglomerates and produced progesterone loaded SLNs of 181 nm or NLCs of 137 nm in diameter. The drug loading of the two formulations was ∼2%. This work showed that the composition of the lipids could be used to control the rate of progesterone release from the nanoparticles; the API was released more slowly when formulated into NLCs (Fig. 5B). The type of lipid nanocarrier used for the progesterone also influenced the penetration into skin (Fig. 5C); the amount of progesterone in the top layer of the skin (the stratum corneum) was higher for SLNs than NLCs (Fig. 5D).^111^ In 2018, Elmowafy demonstrated a NLCs formulation of progesterone with fatty alcohols as the lipids. The NLCs were prepared by high shear homogenisation and the mean diameter of the nanoparticles varied between ∼135–225 nm based on the different fatty alcohols used in the formulation. The drug loading however was not reported. They investigated the permeation across *ex vivo* rabbit duodenum and found enhanced permeation *versus* a suspension of progesterone, demonstrating the potential of this approach for enhancing oral delivery of progesterone.^112^

An NLC formulation of nifedipine, combined with simvastatin (medication to treat elevated cholesterol), has been produced for potential treatment of cardiovascular disease. Hassan *et al.* used a quality by design and a design of experiments approach to optimise the formulation.^113^ The nanomedicine was produced by a combined solvent evaporation and hot homogenisation method. The optimised nanoparticles had a mean diameter of 200 nm, however, it was not clear what drug loading was achieved in this formulation.^113^ This article demonstrated the benefit of using a design of experiments approach; they varied a large number of factors in their formulations to effectively identify the optimal formulation. Something that would have likely been much more time consuming to achieve with a traditional univariant testing method.

Nanoemulsions are the third type of lipid-based nanomedicine, they are nanoscale particles of liquid lipids. Similar to the other lipid-based nanomedicines previously discussed, the dispersed lipid phase is stabilised by stabiliser molecules. There are a few examples of nanoemulsions which have been investigated as carriers for APIs associated with the treatment of preterm birth. Patki *et al.* developed a lipid-based nanoemulsion with OHPC as the API with dimethylacetamide and medium chain triglycerides as the liquid core and Kolliphor HS 15 as the surfactant.^114^ Their system was a self-nanoemulsifying drug delivery system, an isotropic mixture of oil, surfactant and solvent that forms a stable nanoemulsion when dispersed in aqueous media.^121^ Their optimised system possessed a drug loading of 9% wt., had a mean diameter of 50 nm and was absorbed onto a powder of poly(vinyl alcohol) to allow processing into tablets along with other tabletting excipients. The vaginal administration of their nanoemulsion to a LPS infection-induced preterm birth model in mice showed a reduction in the number of mice delivering preterm: 60% compared to 100% for the mice treated with a blank formulation (no API). The mean time to delivery was increased from 14.5 hours for the blank formulation to 20.2 hours for the OHPC containing nanoemulsion.^114^ Much of the same team of researchers led by Guisto have demonstrated another nanoemulsion that used a liquid mixture of triglyceride and the co-solvent dimethylacetamide as the lipid carrier. The API used was a novel compound, a sphingosine kinase (SphK), inhibitor, 4-[[4-(4-chlorophenyl)-2-thiazolyl]amino]phenol, SKI II. SKI II had been shown to inhibit SphK, a key enzyme in the pathways that triggers contraction of myometrial tissue in response to lipopolysaccharide exposure.^122^ SKI II is a lipophilic compound with a solubility in aqueous media of less than 1 μg ml^−1^.^115^ The nanoemulsion was also a self-nanoemulsifying drug-delivery system. The authors studied different compositions of lipid, co-solvent and surfactant in order to determine an optimised nanoemulsion which had a mean diameter of 37 nm.^115^ Upon administration of the SKI II nanoemulsion *via* the vaginal route to LPS-infected mice, there was a significant reduction in rate of preterm birth. This work shows that SKI II could be a useful future option for treating preterm birth triggered by bacterial inflammation. However, SKI II is a compound still in early development and further work is needed to progress the API towards clinical trials.

There are also published examples of the development of nanoemulsions of other APIs with tocolytic properties, although with a focus on use for other clinical indications. For example, indomethacin nanoemulsions have been shown for ocular applications.^116,117^ Klang *et al.* showed that positively charged nanoemulsions of Lipoid E-80 (mainly comprising of phosphatidylcholine) with stearyl amine providing the positive surface charge, produced prolonged duration of the API on the cornea.^116^ Yamaguchi *et al.* produced nanoemulsions of castor oil by high-pressure homogenisation and used chitosan to provide mucoadhesive properties. The resulting nanoparticles had a mean diameter of 117 nm and a drug loading of 0.048% with regards to total solids mass in the formulation. The use of chitosan as a coating enhanced the attachment of the formulation onto mucin and increased the residence time of the indomethacin in the tear fluid.^117^

From the successful encapsulation of APIs with tocolytic properties such as indomethacin and nifedipine as well as progestogens in SLNs, NLCs and nanoemulsions, it is clear that there is also potential for these systems to be engineered specifically for applications in preterm birth (a summary of all the lipid-based nanomedicines can be seen in Table 4). The examples of how stabilisers such as chitosan can be used to alter the biological behaviour demonstrate that lipid-based nanomedicines also have the potential for uterine targeting, thus providing enhanced tocolytic activity. The existing publications on the APIs relevant to preterm birth have shown a number of potential benefits including: (1) the ability to freeze dry and redisperse some lipid-based nanomedicines provides the opportunity to improve formulation storage stability. (2) The composition of the lipids in the nanoparticles can be modified to tune drug release behaviour. (3) The drug loadings of lipid-based nanoparticles are typically ∼2–3% with upper values of 11% reported for NLCs, which is much higher than those reported for liposomes and hence provide greater drug delivery. (4) The ability of lipid-based nanoparticles, particularly NLCs, to enhance permeability across biological barriers may allow the nanomedicines to open new administration routes for these tocolytic APIs.

**Table 4:** Lipid-based nanomedicines containing APIs with tocolytic properties^a^^,^^b^

API	Carrier type	Lipid(s)	Stabiliser(s)	Mean diameter (nm)	Drug loading (wt%)	Administration route	Ref.
Indomethacin	SLNs	Compritol 888 ATO	Tween 80 and poloxamer 188	140	1.9	Topical (ocular)	103
Indomethacin	SLNs	Compritol 888 ATO	Tween 80, poloxamer 188 and chitosan	265	1.8	Topical (ocular)	107
Nifedipine	SLNs	Hydrogenated soybean phosphatidylcholine and dipalmitoylphosphatidyl glycerol	Stabilisation provided by the charge by of the phospholipid	69	2.7	None specified	108
Progesterone	SLNs	2(R)-2,3-dihydroxypropanoate of octadecyl 2,3-dihydroxypropano-ate	Tween 20	640	3	Vaginal	109
Indomethacin	NLCs	Miglyol 812 and Compritol 888 ATO	Tween 80	227	9.9	Topical (ocular)	107
Indomethacin and a non-tocolytic API (celastrol)	NLCs	Precirol ATO 5 (solid lipid) and Labrasol ALF	Cremophor RH40	27	3.6	Transdermal	105
Progesterone	NLCs	Monostearin, stearic acid and oleic acid	Polyethylene glycol	340–385	3–11	Oral	110
			Monostearate and Tween 20				
Progesterone	SLNs	Tristearin	Poloxamer 188	180	2	Topical (skin)	111
Progesterone	NLCs	Tristearin and Miglyol 812 N	Poloxamer 188	140	2	Topical (skin)	111
Progesterone	NLCs	Stearic acid, sesame oil and cetyl alcohol, cetostearyl alcohol	Tween 80 and PEG mixture (400, 1500, 4000)	135–225	*	Oral	112
Nifedipine and a non-tocolytic API (simvastatin)	NLCs	Precirol ATO 5 and Capryol 90	Poloxamer 407, Gelucire 44/14, Tween 20, and lecithin	200	*	Oral	113
OHPC	NE	Dimethylacetamide and medium chain triglyceride	Kolliphor HS 15	50	10	Vaginal	114
SKI II	NE	Dimethylacetamide and medium chain triglyceride	Kolliphor HS 15	37	*	Vaginal	115
Indomethacin	NE	Lipoid E-80	Poloxamer 188 and stearylamine	110	0.1	Topical (ocular)	116
Indomethacin	NE	Castor oil	Polysorbate 80	117	0.05	Topical (ocular)	117

a NE-nanoemulsion.

b * values were not reported in the paper and could not be calculated based on the information given in the experimental.

### Polymer nanoparticles

**Fig. 6:**
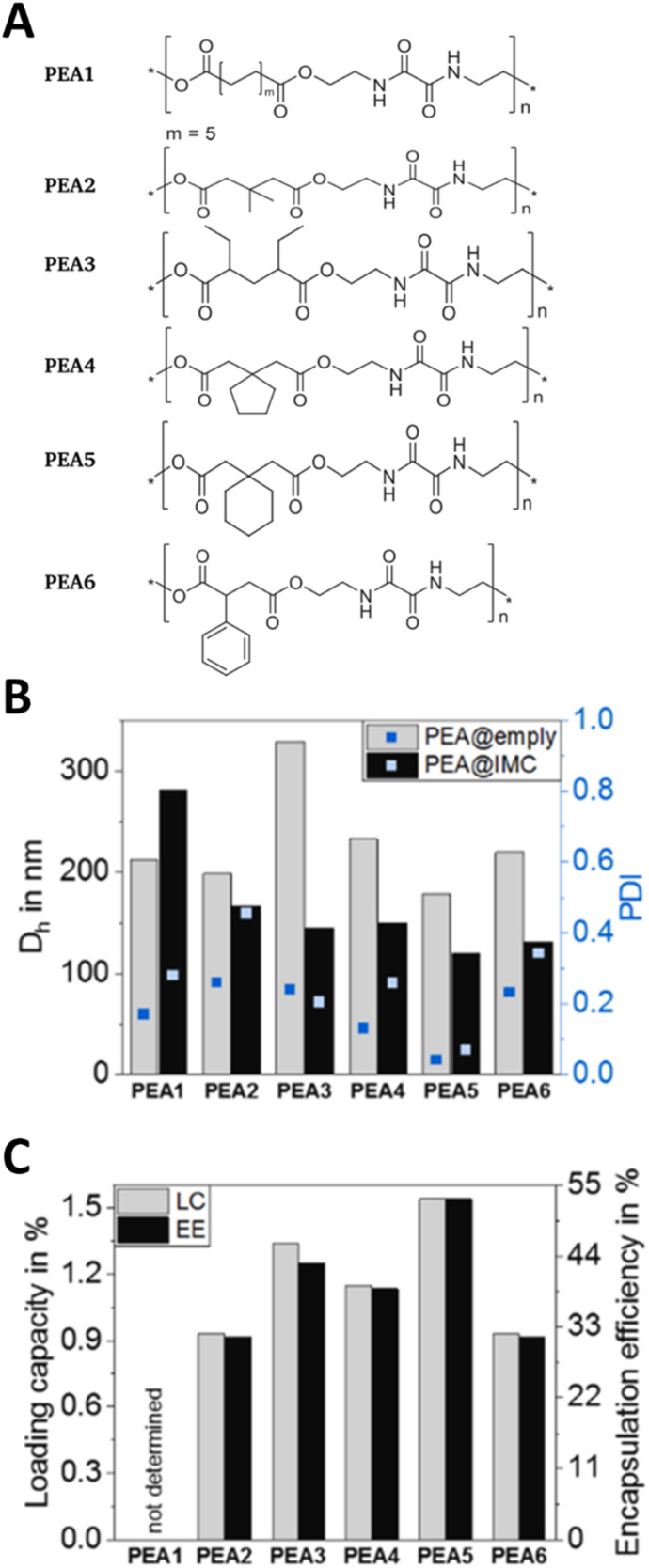
Polymer nanoparticles using poly(ester amide)s nanoparticles and indomethacin at the API. (A) The repeat units of the poly(ester amide)s used in the research. (B) Characterisation of the unloaded poly(ester amide)s nanoparticles (PEA@empty) and indomethacin loaded poly(ester amide)s nanoparticles (PEA@IMC NPs) by DLS to give the mean diameter (Dh) and polydispersity index (PDI). (C) The drug loading capacity (LC) and encapsulation efficiency (EE) of indomethacin loaded poly(ester amide)s nanoparticles. All parts of this figure are taken with permission from Elsevier ref. 126.

Polymer nanoparticles are submicron particles with a core consisting of a hydrophobic (non-water soluble) polymer. These nanoparticles are coated with stabilisers that have a polar character and provide colloidal stability.^123^ The polymer core is made of a degradable polymer to ensure that the nanoparticle does not persist in the body after the drug has been delivered. While a wide range of polymers have been investigated this application, polyesters, specifically polylactic acid (PLA), polyglycolic acid (PGA), their copolymer poly(lactic-co-glycolic acid) (PLGA) and polycaprolactone (PCL) are the most widely used polymers due to their well-documented use in biomedical applications.^124^ Whilst we were not able to find any examples of polymer nanoparticles that had been designed for application in preterm birth *per se*, APIs used to treat preterm birth have been formulated as polymer nanoparticles for use in other indications. For example, Alkholief *et al.* produced a PLGA nanoformulation of indomethacin; they used an emulsion solvent evaporation method to investigate the production parameters using two different surfactants (polyvinylpyrrolidone and polyvinyl alcohol), at a range of concentrations and different organic solvents. Depending on the conditions used, mean particle sizes of ∼280–610 nm were obtained, with higher surfactant concentrations yielding larger particle size. The indomethacin drug loadings were 5–6.8% of the formulations. Cytotoxicity tests showed that encapsulating the indomethacin in the polymer nanoparticles reduced the toxicity in HepG2 liver cells compared to the free indomethacin.^125^ The reduced toxicity of their formulation might have been caused by a reduction in the concentration of free indomethacin, or differences in the accumulation of the nanoparticles into the cells, although accumulation was not investigated. Muljajew *et al.* produced polymer nanoparticles containing indomethacin by a nanoprecipitation approach. They assessed six poly(ester amides) (Fig. 6A) as the carrier and combined a modelling and experimental high throughput screening method to determine the optimal conditions for obtaining indomethacin polymer nanoparticles. Particles with mean diameters of 100–400 nm (Fig. 6B) and modest drug loadings of 0.9–1.5% (Fig. 6C) were obtained.^126^ The combined modelling and high throughput experimental approach may provide an opportunity to develop future nanoformulations in a more informed manner than the traditional screening methods typically used elsewhere; this combination of modelling may also accelerate the development of new formulations.

The potential to produce polymer nanoparticles containing indomethacin is attractive for the treatment of preterm birth, particularly as the examples show that it is possible to obtain higher drug loadings than in liposomes (see Table 5 for a summary of the polymer nanoparticle formulations). Polymer nanoparticles have also been produced with targeting ligands.^127^ Therefore, it is highly feasible to utilise the OTR targeting (or others as they are identified) approaches as shown in liposomes and apply them to polymer nanoparticles for use in the treatment of preterm birth. To further investigate this however, it is imperative that the biodistribution and efficacy of the nanomedicines *in vivo* is examined.

**Table 5:** Polymer nanomedicines containing APIs with tocolytic properties

API	Polymer	Stabiliser(s)	Mean diameter (nm)	Drug loading (wt%)	Administration route	Ref
Indomethacin	PLGA	Polyvinylpyrrolidone and polyvinyl alcohol	280–610	5–6.8	Not specified	125
Indomethacin	Poly(ester amides) (repeat units can be seen in Fig. 6A)	No surfactant or polyvinyl alcohol	100–400	0.9–1.5	Not specified	126

### Nanosuspensions

Nanosuspensions, also known as nanocrystals or solid drug nanoparticles, are sub-micron particles of water insoluble (high log *P*) compounds. The hydrophobic surfaces of the particles are coated by the adsorption of stabilisers which provide colloidal stability.^128^ Nanosuspensions differ from the other nanomedicines in that they do not contain a carrier material, rather the nanoparticles are themselves composed entirely of the poorly water-soluble API. This means that nanosuspensions offer the highest drug loadings out of all nanomedicines. Nanosuspensions of a wide range of poorly water-soluble APIs can be produced *via* a number of routes, with milling,^129–131^ nanoprecipitation,^132–134^ high pressure homogenisation^135^ and emulsion-templated freeze drying^136–139^ being some of the most common methods.

**Fig. 7:**
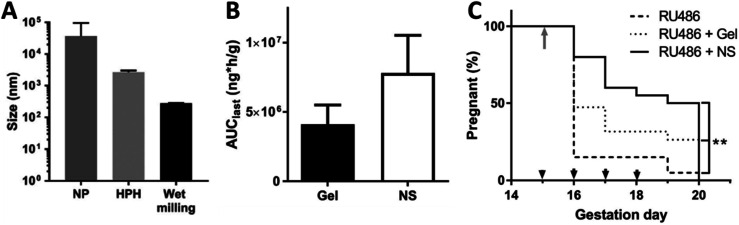
Nanosuspensions containing progesterone investigated for the treatment of preterm birth. (A) Mean diameter of progesterone nanosuspension produced by different size reduction techniques, nanoprecipitation (NP), high pressure homogenisation (HPH) and wet milling (the wet milling duration used was 9 h). (B) Area under the curve (AUC) in the cervix after 24 h (AUClast) after a single vaginal dose in healthy pregnant mice on gestation day 15. (C) Comparison of the percentage of animals remaining pregnant after RU486 injection on gestation day 15 (grey arrow) then treated with either the progesterone nanosuspension (NS) or the progesterone gel. Animals in the treatment groups received daily vaginal doses from (black arrowheads). ** denotes log-rank *p* = 0.001 for all curve comparison. All parts of the figure are adapted with permission from Elsevier ref. 140.

Hoang *et al.* reported the successful formulation of a nanosuspension of progesterone. Nanosuspensions were prepared by three different processing methods, nanoprecipitation, high pressure homogenisation and wet milling, all using poloxamer 407 (Pluronic® F127) as the stabiliser. Wet milling produced the smallest particle size with a mean diameter of ∼260 nm (Fig. 7A). The drug loading in the formulation was not given but we calculated it to be 80%. The authors carried out an *in vivo* comparison of their nanosuspension formulation to a gel formulation of progesterone (Crinone® 8%). They showed that when administered vaginally the gel formulation displayed significantly less progesterone uptake in the cervix compared to a nanosuspension formulation of progesterone. The maximum concentration (*C*_max_) of progesterone in the cervix using the nanosuspension was increased 5-fold compared to the branded gel (Fig. 7B). The authors speculated that the improvement in drug delivery was due to particle size and the PEG coating which made the nanoparticles mucoinert and therefore able to better penetrate through the mucus lining of the cervix. In an *in vivo* trial of progesterone rescue in mice where preterm birth was initiated using a progesterone antagonist, RU486, the nanosuspension increased the number of pups delivering full term compared to the commercial progesterone gel (Fig. 7C).^140^ Further work by ensign exploring nanosuspensions for the treatment of preterm birth, has focussed on the use of histone deacetylase (HDAC) inhibitors.^141^ Histone deacetylation has been found to increase at term in both the myometrium (smooth muscle of the uterus) and the cervix. Trichostatin A is a potent HDAC inhibitor. Recently, Zierden *et al.* produced a nanosuspension formulation by milling trichostatin A with Pluronic® F127 to give a mean diameter of ∼200 nm as measured by DLS. They investigated the vaginal delivery of a combination of the trichostatin A and progesterone nanosuspensions (in 1.5 : 100 mass ratio of the two APIs) in an LPS-induced mouse model. The combination treatment was more effective at preventing preterm birth than either of the nanosuspensions independently. They also found combined application of the two APIs inhibited cell contractility in human myometrial cells *in vitro*.^141^

A nanosuspension of indomethacin has been produced by Styliari *et al.* by using block copolymer of PEG and PCL (PEG–PCL) as the polymer stabiliser.^142^ They combined molecular simulations with experimental testing to investigate how different PEG chain lengths of block copolymers of PEG-PCL controlled the formation of polymer coated nanosuspensions using a nanoprecipitation type method. In this work, the indomethacin was processed as a coarse aqueous nanosuspension by sonication (mean diameter by DLS of ∼700 nm), then this suspension was mixed with an acetone solution of PEG–PCL in a T-piece. The authors hypothesised that the acetone solution of the polymer would partially dissolve the larger nanoparticles of indomethacin and also provide colloidal stabilisation. They observed a reduction in particle diameter, obtaining particles of ∼250 nm diameter, and drug loadings of up to 78% were also reported.

Nanosuspensions offer the highest drug loadings out of all the nanomedicine options discussed in this review, with values of 80% reported (see Table 6 for a summary of the nanosuspension formulations). This attribute of high drug loadings for nanosuspensions is very useful for APIs with lower potency values, where considerable amounts of a nanomedicine may need to be administered to achieve a therapeutic dose. Furthermore, there are many examples of nanosuspensions that can be processed into a dry form that can be redispersed upon use, and hence overcome some storage stability issues.^137,143,144^ While examples of targeted nanosuspensions are less common than other nanomedicines, progress has been made in this area with the design of targeting ligands that can adsorb onto the surface of the nanoparticles.^145^

**Table 6:** Nanosuspension nanomedicines containing APIs with tocolytic properties^a^

API	Stabiliser	Mean diameter (nm)	Drug loading (wt%)	Administration route	Ref.
Progesterone	Pluronic F127	260	80	Vaginal	140
Trichostatin A	Pluronic F127	200	*	Vaginal	141
Indomethacin	PEG-PCL	250	78	Not specified	142

a * values were not reported in the paper and could not be calculated based on the information given in the experimental.

## Conclusions and future work

Preterm birth represents a major, pressing, global healthcare problem with an urgent need for more effective treatments. New formulations of APIs are required to prolong gestation, to improve dosage profiles, enhance targeting to the uterine tissue, reduce feto-maternal side effects and ultimately improve safety and efficacy. The use of nanomedicine for the delivery of tocolytics is still at an early stage; the first example of a nanomedicine delivering a tocolytic *in vivo* was published in 2015.^85^ Over the past 8 years there has been considerable improvements in nanomedicine technology for tocolytic therapy, this has been led by Refuerzo, Paul and Hua showing promising manipulation of several tocolytics in liposomal formulations.^76,88,90^ Collectively, their key findings have shown increased uterine targeting, increased uterine uptake, reduced placental transfer and a noteworthy improvement on uterine quiescence with concomitant reduction in preterm birth delivery rates in *in vivo* mouse models. Liposomes are likely the nanomedicine type that is most progressed in the field of preterm birth because liposomes were amongst the first approved nanomedicines; liposomal doxorubicin (a cancer drug) was approved by the FDA in 1995.^146^ This success de-risked the development of liposomal formulations; the majority of the earliest clinically approved nanomedicines were liposomes,^147^ and liposomes initially dominated early clinical nanomedicine success.^148^ However, more recently other nanomedicine types have now moved through the development pathway with 14% and 8% of clinical trials in investigating lipid nanoparticles and polymer nanoparticles respectively. This is a considerable growth in the development of such systems as lipid nanoparticles and polymer nanoparticles only represent ∼3% and 0% of currently approved nanomedicines.^148^ While liposomes are attractive nanocarriers they tend to be less stable than the other nanomedicine types, and are not typically suitable for oral dosing due to degradation under acidic conditions.^149^ As such, the opportunities offered by other nanomedicine types other than liposomes should be more effectively explored for preterm birth. As identified in this review, there are many examples of other lipid-based nanomedicines where tocolytic APIs could be formulated to potentially provide increased drug loading compared to liposomes. Such lipid-based nanomedicines can use the same approaches to incorporating targetting as liposomes, such as the use of lipid-PEG-ligand conjugates. Therefore, the successful targeting identified with liposomes can likely be adapted and used for lipid-based nanoparticles. Polymer nanoparticles are the nanomedicine type that has the fewest examples of the delivery of tocolytic APIs. However, this should not exclude polymer nanoparticles as a potential opportunity, they have good stability during preparation and storage in biological fluids. Additionally, polymer nanoparticles have been shown to offer many of the benefits of nanomedicine types (targeting, the ability to tune size and surface chemistry, *etc.*)^150^ which should allow application in the treatment of preterm birth. Finally, nanosuspensions offer a route to deliver high concentrations of the API due to their very high drug loadings of up to ∼80% wt relative to excipients. Nanosuspensions are the second most progressed nanomedicine type (after liposomes) for the treatment of preterm birth with *in vivo* trials showing significantly more efficient in drug delivery for a nanosuspension of progesterone than unformulated progesterone. There is therefore a considerable potential for non-lipid systems to also be used as potential tocolytic therapeutics. Additionally, repurposing nanomedicine formulations from other indications may provide a considerable opportunity to diversify the range of formulations available for application in preterm birth.

Nanomedicines can be produced with many different particle properties including: size, surface charge, surface chemistry and carrier composition, all of which can considerably affect biological behaviour. The studies that show the most positive results in terms of reduction in preterm birth in mice have been using liposomes ∼200 nm in diameter for IV dosing or 200 nm nanosuspensions for vaginal dosing of progesterone. For IV dosing, targeting is critical to achieve a therapeutic effect, however with regards to the other particle properties there have not been studies to ascertain the optimal parameters. In terms of nanomedicine size, studies on the biodistribution of nanoparticles in pregnant animals have shown that there is a considerable difference in the maternal-to-foetal transfer based on the type and size of nanoparticles. Such analysis indicates that smaller nanoparticles (less than ∼10 nm) may be more likely to enter the foetus.^90^ However, there is not the data to generalise if this is true across all nanomedicine types. Furthermore, the density of the polymer coating on the nanoparticle may considerably alter the ability of nanoparticles to penetrate through tissue. For example, the use of a dense coating of PEG has been found to facilitate an increase in the mobility of polymer nanoparticles in tissue.^151^ Additionally, studies on targeted nanomedicines for other applications have shown that factors such as the density of the targetting ligand and the length of the polymer used between the nanoparticle and the targetting ligand can play a key role in influencing the targeting efficacy.^152,153^ Therefore, the careful selection of the polymer stabiliser is a key consideration for targeted nanomedicines as it has the potential to alter targeting efficacy or potential lead to increased transfer to the fetus. Currently, these knowledge gaps make it challenging to efficiently design nanomedicines for the treatment of preterm birth based on IV dosing. For vaginal dosing of nanomedicines with the aim to avoid mucoadhesion, there is data to support some elements of the nanomedicine design. Trends for enhancing muco-penetration have been shown across at least two nanoparticle types, with both polymer nanoparticles^75,154^ and nanosuspensions^140^ showing that particles with mean diameters in the range 110–260 nm and coated with polymers that provided a PEG coating have enhanced muco-penetration. In the future, the production of nanomedicines with systematic structural variation would allow more detailed studies in the relationships between particle properties and biological behaviour. Identifying the key design rules for nanomedicines for the treatment of preterm birth would considerably enhance progress in this field.

The drug loading of an API within a nanomedicine is another key factor that needs to be considered in order to allow clinical translation of novel formulations. With the exception of the nanosuspensions, many of the nanomedicines report API loadings of ∼1–4%. If we focus on indomethacin, the most widely investigated nanomedicine formulations of a tocolytic API, it is typically given orally in doses of 25–100 mg as a tocolytic and has a bioavailability of virtually 100%.^155^ Assuming that a similar IV dose of indomethacin would be used in a new nanomedicine formulation with a 1% drug loading, then potentially 2.5–10.0 g of a nanomedicine formulation would need to be infused. This amount of excipients in the formulation is much larger compared to other clinically used liposome systems such as liposomal doxorubicin (which has a API loading of 16.7%).^156^ Further optimisation of the current liposome formulations or use of higher drug loading lipid-based nanomedicines such as NLCs may address this issue.

From a clinical perspective, future developments in nanomedicines for the prevention of preterm birth are likely to be beneficial in the acute setting of clinical management of women in threatened spontaneous labour. Preterm birth is an attractive target due to the very high risks of morbidity and mortality to the neonate. As such, preterm birth is associated with a very significant financial cost both in the acute hospital setting of neonatal intensive care unit and to individuals and society in the longer term from caring for infants harmed by preterm birth (neurodevelopmental delay *etc.*). Women in preterm labour currently receive IV medications such as magnesium sulfate, antibiotics and tocolytics. As such, the administration of nanomedicine by IV should be suitable for a clinical environment. The clinical application of nanomedicines is likely to be beneficial in the acute setting to allow reduction in dosing regimen to prevent side effects. Additionally, improvements in clinical management of preterm birth from the cessation of uterine activity would allow transfer to specialist neonatal facilities, administration of antenatal corticosteroids or potentially the prolongation of pregnancy all of which have strong associations with improved neonatal outcomes.

Ultimately, the treatment of preterm birth is a considerable opportunity for nanomedicine to address, improved therapies would offer significant health benefits globally. Addressing the challenges identified in this review should allow for the accelerated development of nanomedicine technologies that have a greater potential for clinical translation.

## Conflicts of interest

SR is a Director of Tandem Nano Ltd and co-inventor of patents relating to drug delivery using nanotechnologies. SR has received research funding from GSK/ViiV, AZ and Gilead and a consultancy from Gilead. TM is a co-inventor of patents relating to drug delivery using nanotechnologies and has carried out consultancy work for Vifor Pharma.

## Supplementary Material

## References

[cit1] World Health Organization (WHO) , New Global Estimates on Preterm Birth, 2018

[cit2] Liu L., Oza S., Hogan D., Chu Y., Perin J., Zhu J., Lawn J. E., Cousens S., Mathers C., Black R. E. (2016). Lancet.

[cit3] Walani S. R. (2020). Internet J. Gynecol. Obstet..

[cit4] Chawanpaiboon S., Vogel J. P., Moller A. B., Lumbiganon P., Petzold M., Hogan D., Landoulsi S., Jampathong N., Kongwattanakul K., Laopaiboon M., Lewis C., Rattanakanokchai S., Teng D. N., Thinkhamrop J., Watananirun K., Zhang J., Zhou W., Gülmezoglu A. M. (2019). Lancet Global Health.

[cit5] Engle W. A., Tomashek K. M., Wallman C. (2007). Pediatrics.

[cit6] Al-Alaiyan S. (2008). Ann. Saudi Med..

[cit7] Macfarlane P. I., Wood S., Bennett J. (2003). Arch. Dis. Child. Fetal Neonatal Ed..

[cit8] Larroque P. T. B., Breart G., Kaminski M., Dehan M., Andre M., Burguet A., Grandjean H., Ledesert B., Leveque C., Maillard F., Matis J., Roze J. (2004). Arch. Dis. Child. Fetal Neonatal Ed..

[cit9] Chatterjee J., Gullam J., Vatish M., Thornton S. (2007). Arch. Dis. Child. Fetal Neonatal Ed..

[cit10] Lozano R., Naghavi M., Foreman K., Lim S., Shibuya K., Aboyans V., Abraham J., Adair T., Aggarwal R., Ahn S. Y., AlMazroa M. A., Alvarado M., Anderson H. R., Anderson L. M., Andrews K. G., Atkinson C., Baddour L. M., Barker-Collo S., Bartels D. H., Bell M. L., Benjamin E. J., Bennett D., Bhalla K., Bikbov B., Bin Abdulhak A., Birbeck G., Blyth F., Bolliger I., Boufous S., Bucello C., Burch M., Burney P., Carapetis J., Chen H., Chou D., Chugh S. S., Coffeng L. E., Colan S. D., Colquhoun S., Colson K. E., Condon J., Connor M. D., Cooper L. T., Corriere M., Cortinovis M., Courville De Vaccaro K., Couser W., Cowie B. C., Criqui M. H., Cross M., Dabhadkar K. C., Dahodwala N., De Leo D., Degenhardt L., Delossantos A., Denenberg J., Des Jarlais D. C., Dharmaratne S. D., Dorsey E. R., Driscoll T., Duber H., Ebel B., Erwin P. J., Espindola P., Ezzati M., Feigin V., Flaxman A. D., Forouzanfar M. H., Fowkes F. G. R., Franklin R., Fransen M., Freeman M. K., Gabriel S. E., Gakidou E., Gaspari F., Gillum R. F., Gonzalez-Medina D., Halasa Y. A., Haring D., Harrison J. E., Havmoeller R., Hay R. J., Hoen B., Hotez P. J., Hoy D., Jacobsen K. H., James S. L., Jasrasaria R., Jayaraman S., Johns N., Karthikeyan G., Kassebaum N., Keren A., Khoo J. P., Knowlton L. M., Kobusingye O., Koranteng A., Krishnamurthi R., Lipnick M., Lipshultz S. E., Lockett Ohno S., Mabweijano J., MacIntyre M. F., Mallinger L., March L., Marks G. B., Marks R., Matsumori A., Matzopoulos R., Mayosi B. M., McAnulty J. H., McDermott M. M., McGrath J., Memish Z. A., Mensah G. A., Merriman T. R., Michaud C., Miller M., Miller T. R., Mock C., Mocumbi A. O., Mokdad A. A., Moran A., Mulholland K., Nair M. N., Naldi L., Narayan K. M. V., Nasseri K., Norman P., O'Donnell M., Omer S. B., Ortblad K., Osborne R., Ozgediz D., Pahari B., Pandian J. D., Panozo Rivero A., Perez Padilla R., Perez-Ruiz F., Perico N., Phillips D., Pierce K., Pope C. A., Porrini E., Pourmalek F., Raju M., Ranganathan D., Rehm J. T., Rein D. B., Remuzzi G., Rivara F. P., Roberts T., Rodriguez De León F., Rosenfeld L. C., Rushton L., Sacco R. L., Salomon J. A., Sampson U., Sanman E., Schwebel D. C., Segui-Gomez M., Shepard D. S., Singh D., Singleton J., Sliwa K., Smith E., Steer A., Taylor J. A., Thomas B., Tleyjeh I. M., Towbin J. A., Truelsen T., Undurraga E. A., Venketasubramanian N., Vijayakumar L., Vos T., Wagner G. R., Wang M., Wang W., Watt K., Weinstock M. A., Weintraub R., Wilkinson J. D., Woolf A. D., Wulf S., Yeh P. H., Yip P., Zabetian A., Zheng Z. J., Lopez A. D., Murray C. J. L. (2012). Lancet.

[cit11] National Institute for Health and Care Excellence (NICE) , Preterm Labour and Birth, 2016 31971700

[cit12] Lightstone L. (2015). Med..

[cit13] nan Zeng L., li Zhang L., Shi J., ling Gu L., Grogan W., Gargano M. M., Chen C. (2014). Taiwan. J. Obstet. Gynecol..

[cit14] Bhattacharjee E., Maitra A. (2021). npj Genomic Med..

[cit15] Ubaldi F. M., Cimadomo D., Vaiarelli A., Fabozzi G., Venturella R., Maggiulli R., Mazzilli R., Ferrero S., Palagiano A., Rienzi L. (2019). Front. Endocrinol..

[cit16] Montgomery K. S., Cubera S., Belcher C., Patrick D., Funderburk H., Melton C., Fastenau M. (2005). J. Perinat. Educ..

[cit17] Fuchs F. S. M. (2016). Semin. Fetal Neonat. Med..

[cit18] Puccio G., Giuffré M., Piccione M., Piro E., Malerba V., Corsello G. (2014). Ital. J. Pediatr..

[cit19] Muhlhausler B. S., Hancock S. N., Bloomfield F. H., Harding R. (2011). Pediatr. Res..

[cit20] Hediger M. L., Scholl T. O., Schall J. I., Krueger P. M. (1997). Ann. Epidemiol..

[cit21] Glick I., Kadish E., Rottenstreich M. (2021). Int. J. Women's Health.

[cit22] Sha T., Yin X., Cheng W., Massey I. Y. (2018). Fertil. Steril..

[cit23] Norman J. E., Marlow N., Messow C. M., Shennan A., Bennett P. R., Thornton S., Robson S. C., McConnachie A., Petrou S., Sebire N. J., Lavender T., Whyte S., Norrie J. (2016). Lancet.

[cit24] Care A., Nevitt S. J., Medley N., Donegan S., Good L., Hampson L., Tudur Smith C., Alfirevic Z. (2022). BMJ.

[cit25] Gaiser R., Meis P. J., Klebanoff M., Thom E., Dombrowski M. P., Sibai B., Moawad A. H., Spong C. Y., Hauth J. C., Miodovnik M., Varner M. W., Leveno K. J., Caritis S. N., Iams J. D., Wapner R. J., Conway D., O'Sullivan M. J., Carpenter M., Mercer B., Ramin S. M., Thorp J. M., Peaceman A. M. (2003). N. Engl. J. Med..

[cit26] Blackwell S. C., Chauhan S. P., Gyamfi-Bannerman C., Biggio J. R., Hughes B. L., Louis J. M., Manuck T. A., Miller H. S., Das A. F., Saade G. R., Nielsen P., Baker J., Yuzko O. M., Reznichenko G. I., Reznichenko N. Y., Pekarev O., Tatarova N., Gudeman J., Duncan M., Williams L., Krop J., Birch R., Jozwiakowski M. J. (2020). Am. J. Perinatol..

[cit27] Murray S. R., Stock S. J., Cowan S., Cooper E. S., Norman J. E. (2018). Obstet. Gynecol..

[cit28] Dodd J. M., Grivell R. M., OBrien C. M., Dowswell T., Deussen A. R. (2019). Cochrane Database Syst. Rev..

[cit29] Stewart L. A., Simmonds M., Duley L., Llewellyn A., Sharif S., Walker R. A., Beresford L., Wright K., Aboulghar M. M., Alfirevic Z., Azargoon A., Bagga R., Bahrami E., Blackwell S. C., Caritis S. N., Combs C. A., Croswell J. M., Crowther C. A., Das A. F., Dickersin K., Dietz K. C., Elimian A., Grobman W. A., Hodkinson A., Maurel K. A., McKenna D. S., Mol B. W., Moley K., Mueller J., Nassar A., Norman J. E., Norrie J., O'Brien J. M., Porcher R., Rajaram S., Rode L., Rouse D. J., Sakala C., Schuit E., Senat M. V., Simpson J. L., Smith K., Tabor A., Thom E. A., van Os M. A., Whitlock E. P., Wood S., Walley T. (2021). Lancet.

[cit30] Walter S., Rehal A., Benkő Z., De Paco Matallana C., Syngelaki A., Janga D., Cicero S., Akolekar R., Singh M., Chaveeva P., Burgos J., Molina F. S., Savvidou M., De La Calle M., Persico N., Quezada Rojas M. S., Sau A., Greco E., O'Gorman N., Plasencia W., Pereira S., Jani J. C., Valino N., del Mar Gil M., Maclagan K., Wright A., Wright D., Nicolaides K. H. (2021). Am. J. Obstet. Gynecol..

[cit31] Romero R., Conde-Agudelo A., Rehal A., Da Fonseca E., Brizot M. L., Rode L., Serra V., Cetingoz E., Syngelaki A., Tabor A., Perales A., Hassan S. S., Nicolaides K. H. (2022). Ultrasound Obstet. Gynecol..

[cit32] Senat M. V., Porcher R., Winer N., Vayssière C., Deruelle P., Capelle M., Bretelle F., Perrotin F., Laurent Y., Connan L., Langer B., Mantel A., Azimi S., Rozenberg P. (2013). Am. J. Obstet. Gynecol..

[cit33] National Institute for Health and Care Excellence (NICE) , Twin and triplet pregnancy, https://www.nice.org.uk/guidance/ng137/chapter/recommendations, (accessed January 2023) 31513365

[cit34] Wilson A., Hodgetts-Morton V. A., Marson E. J., Markland A. D., Larkai E., Papadopoulou A., Coomarasamy A., Tobias A., Chou D., Oladapo O. T., Price M. J., Morris K., Gallos I. D. (2022). Cochrane Database Syst. Rev..

[cit35] Yamasmit W., Chaithongwongwatthana S., Tolosa J. E., Limpongsanurak S., Pereira L., Lumbiganon P. (2015). Cochrane Database Syst. Rev..

[cit36] Derbent A., Simavli S., Gümüş I. I., Tatli M. M., Turhan N. Ö. (2011). Arch. Gynecol. Obstet..

[cit37] Haas D., Caldwell D. M., Kirkpatrick P., McIntosh J. J., Welton N. J. (2012). BMJ.

[cit38] Dodd J. M., Jones L., Flenady V., Crowther C. A. (2013). Cochrane Database Syst. Rev..

[cit39] Cuppett C. D. and Caritis S. N., Uterine Contraction Agents and Tocolytics, Elsevier Inc., 2013

[cit40] Vause S., Johnston T. (2000). Arch. Dis. Child. Fetal Neonatal Ed..

[cit41] Carlin A., Norman J., Cole S., Smith R. (2009). Br. Med. J..

[cit42] Haas D. M., Benjamin T., Sawyer R., Quinney S. K. (2014). Int. J. Women's Health.

[cit43] Hanley M., Sayres L., Reiff E., Wood A., Grotegut C., Kuller J. (2019). Obstet. Gynecol. Surv..

[cit44] Arrowsmith S., Kendrick A., Wray S. (2010). Obstet., Gynaecol. Reprod. Med..

[cit45] Wray S., Arrowsmith S., Sharp A. (2023). Annu. Rev. Pharmacol. Toxicol..

[cit46] Tan T. C., Devendra K., Tan L. K., Tan H. K. (2006). Singapore Med. J..

[cit47] Klumper J., Breebaart W., Roos C., Naaktgeboren C. A., Van Der Post J., Bosmans J., Van Kaam A., Schuit E., Mol B. W., Baalman J., McAuliffe F., Thornton J., Kok M., Oudijk M. A. (2019). BMJ Open.

[cit48] Chan L. W., Sahota D. S., Yeung S. Y., Leung T. Y., Fung T. Y., Lau T. K., Leung T. N. (2008). Hong Kong Med. J..

[cit49] Pryde P. G., Besinger R. E., Gianopoulos J. G., Mittendorf R. (2001). Semin. Perinatol..

[cit50] Gáspár R., Hajagos-tóth J. (2013). Pharmaceuticals.

[cit51] V Tetko I., Gasteiger J., Todeschini R., Mauri A., Livingstone D., Ertl P., Palyulin V. A., V Radchenko E., Zefirov N. S., Makarenko A. S., Tanchuk V. Y., Prokopenko V. V. (2005). J. Comput.-Aided Mol. Des..

[cit52] Jacquemyn Y. (2006). BJOG: Int. J. Obstet. Gynaecol..

[cit53] Fisk N. M., Chan J. (2003). BJOG: Int. J. Obstet. Gynaecol..

[cit54] King J. F. (2004). Curr. Opin. Obstet. Gynecol..

[cit55] Flenady V., Am W., Dnm P., Om S., Murray L., La J., Carbonne B., Flenady V., Am W., Dnm P., Om S., Murray L., La J., Carbonne B. (2014). Cochrane Database Syst. Rev..

[cit56] Economy K. E., Abuhamad A. Z. (2001). Semin. Perinatol..

[cit57] Hammerman C., Glaser J., Kaplan M., Schimmel M. S., Ferber B., Eidelman A. I. (1998). Pediatrics.

[cit58] Hammers A. L., Sanchez-Ramos L., Kaunitz A. M. (2015). Am. J. Obstet. Gynecol..

[cit59] Zierden H. C., Shapiro R. L., DeLong K., Carter D. M., Ensign L. M. (2021). Adv. Drug Delivery Rev..

[cit60] Kammari R. , Das N. G. and Das S. K., Nanoparticulate Systems for Therapeutic and Diagnostic Applications, Elsevier, 2017

[cit61] Mirza A. Z., Siddiqui F. A. (2014). Int. Nano Lett..

[cit62] Soares S., Sousa J., Pais A., Vitorino C. (2018). Front. Chem..

[cit63] Olusanya T. O. B., Ahmad R. R. H., Ibegbu D. M., Smith J. R., Elkordy A. A. (2018). Molecules.

[cit64] Nehoff H., Parayath N. N., Domanovitch L., Taurin S., Greish K. (2014). Int. J. Nanomed..

[cit65] Bazak R., Houri M., El Achy S., Hussein W., Refaat T. (2014). Mol. Clin. Oncol..

[cit66] Wicki A., Witzigmann D., Balasubramanian V., Huwyler J. (2015). J. Controlled Release.

[cit67] Lipinski C. A. (2004). Drug Discovery Today: Technol..

[cit68] Ghadi R., Dand N. (2017). J. Controlled Release.

[cit69] Jacob S., Nair A. B., Shah J. (2020). Biomater. Res..

[cit70] Talegaonkar S., Bhattacharyya A. (2019). AAPS PharmSciTech.

[cit71] Parodi A., Buzaeva P., Nigovora D., Baldin A., Kostyushev D., Chulanov V., Savvateeva L. V., Zamyatnin A. A. (2021). J. Nanobiotechnol..

[cit72] Alexander N. J., Baker E., Kaptein M., Karck U., Miller L., Zampaglione E. (2004). Fertil. Steril..

[cit73] Einer-Jensen N., Cicinelli E., Galantino P., Pinto V., Barba B. (2002). Hum. Reprod..

[cit74] Mir A., Vartak R. V., Patel K., Yellon S. M., Reznik S. E. (2022). Pharmaceutics.

[cit75] Ensign L. M., Tang B. C., Wang Y. Y., Tse T. A., Hoen T., Cone R., Hanes J. (2012). Sci. Transl. Med..

[cit76] Refuerzo J. S., Leonard F., Bulayeva N., Gorenstein D., Chiossi G., Ontiveros A., Longo M., Godin B. (2016). Sci. Rep..

[cit77] Mitchell M. J., Billingsley M. M., Haley R. M., Wechsler M. E., Peppas N. A., Langer R. (2021). Nat. Rev. Drug Discovery.

[cit78] Albanese A., Tang P. S., Chan W. C. W. (2012). Annu. Rev. Biomed. Eng..

[cit79] Taha M. S., Padmakumar S., Singh A., Amiji M. M. (2020). Drug Deliv. Transl. Res..

[cit80] Wong J., Brugger A., Khare A., Chaubal M., Papadopoulos P., Rabinow B., Kipp J., Ning J. (2008). Adv. Drug Delivery Rev..

[cit81] Danaei M., Dehghankhold M., Ataei S., Hasanzadeh Davarani F., Javanmard R., Dokhani A., Khorasani S., Mozafari M. R. (2018). Pharmaceutics.

[cit82] Wang Y., Pi C., Feng X., Hou Y., Zhao L., Wei Y. (2020). Int. J. Nanomed..

[cit83] Poley M., Mora-Raimundo P., Shammai Y., Kaduri M., Koren L., Adir O., Shklover J., Shainsky-Roitman J., Ramishetti S., Man F., De Rosales R. T. M., Zinger A., Peer D., Ben-Aharon I., Schroeder A. (2022). ACS Nano.

[cit84] Wagner A., Vorauer-Uhl K. (2011). J. Drug Delivery.

[cit85] Refuerzo J. S., Alexander J. F., Leonard F., Leon M., Longo M., Godin B. (2015). Am. J. Obstet. Gynecol..

[cit86] Paul J. W., Hua S., Ilicic M., Tolosa J. M., Butler T., Robertson S., Smith R. (2017). Am. J. Obstet. Gynecol..

[cit87] Hua S., Chang H.-I. I., Davies N. M., Cabot P. J. (2011). J. Liposome Res..

[cit88] Hua S., Vaughan B. (2019). Int. J. Nanomed..

[cit89] Hua S. (2019). J. Liposome Res..

[cit90] Paul J. W., Smith R. (2018). Best Pract. Res., Clin. Obstet. Gynaecol..

[cit91] Fischer D. P., Griffiths A. L., Lui S., Sabar U. J., Farrar D., O'Donovan P. J., Woodward D. F., Marshall K. M. (2020). J. Pharmacol. Exp. Ther..

[cit92] Sercombe L., Veerati T., Moheimani F., Wu S. Y., Sood A. K., Hua S. (2015). Front. Pharmacol..

[cit93] Samimi S. , Maghsoudnia N., Eftekhari R. B. and Dorkoosh F., Lipid-Based Nanoparticles for Drug Delivery Systems, Elsevier Inc., 2018

[cit94] Muller R. H., Mader K., Gohla S. (2000). Eur. J. Pharm. Biopharm..

[cit95] Pardeshi C., Rajput P., Belgamwar V., Tekade A., Patil G., Chaudhary K., Sonje A. (2012). Acta Pharm.

[cit96] Mukherjee S., Ray S., Thakur R. S. (2009). Indian J. Pharm. Sci..

[cit97] Ekambaram P., Sathali A. A. H., Priyanka K. (2018). Sci. Rev. Chem. Commun..

[cit98] Madan J. R., Khude P. A., Dua K. (2014). Int. J. Pharm. Invest..

[cit99] Shtay R., Ping C., Schwarz K. (2018). J. Food Eng..

[cit100] Zhu Z. (2014). Mol. Pharm..

[cit101] Hogarth C., Arnold K., McLauchlin A., Rannard S. P., Siccardi M., McDonald T. O. (2021). J. Mater. Chem. B.

[cit102] Taylor J. M., Scale K., Arrowsmith S., Sharp A., Flynn S., Rannard S., Mcdonald T. O. (2020). Nanoscale.

[cit103] Hippalgaonkar K. K., Adelli G. R., Hippalgaonkar K. K., Repka M. A., Majumdar S. (2013). J. Ocul. Pharmacol. Ther..

[cit104] Bayón-Cordero L., Alkorta I., Arana L. (2019). Nanomaterials.

[cit105] Kang Q., Liu J., Zhao Y., Liu X. Y., Liu X. Y., Wang Y. J., Mo N. L., Wu Q. (2018). Artif. Cells, Nanomed., Biotechnol..

[cit106] Mohammed M. A., Syeda J. T. M., Wasan K. M., Wasan E. K. (2017). Pharmaceutics.

[cit107] Balguri S. P., Adelli G. R., Majumdar S. (2016). Eur. J. Pharm. Biopharm..

[cit108] Barman R. K., Iwao Y., Funakoshi Y., Ranneh A. H., Noguchi S., Ibne Wahed M. I., Itai S. (2014). Chem. Pharm. Bull..

[cit109] Cassano R., Trombino S. (2019). Int. J. Polym. Sci..

[cit110] Yuan H., Wang L. L., Du Y. Z., You J., Hu F. Q., Zeng S. (2007). Colloids Surf., B.

[cit111] Esposito E., Sguizzato M., Drechsler M., Mariani P., Carducci F., Nastruzzi C., Cortesi R. (2017). Eur. J. Pharm. Biopharm..

[cit112] Elmowafy M., Shalaby K., Badran M. M., Ali H. M., Abdel-Bakky M. S., El-Bagory I. (2018). J. Drug Delivery Sci. Technol..

[cit113] Hassan T. H., Salman S. S., Elkhoudary M. M., Gad S. (2021). J. Drug Delivery Sci. Technol..

[cit114] Patki M., Giusto K., Gorasiya S., Reznik S. E., Patel K. (2019). Pharmaceutics.

[cit115] Giusto K., Patki M., Koya J., Ashby C. R., Munnangi S., Patel K., Reznik S. E. (2019). Nanomedicine.

[cit116] Klang S., Abdulrazik M., Benita S. (2000). Pharm. Dev. Technol..

[cit117] Yamaguchi M., Ueda K., Isowaki A., Ohtori A., Takeuchi H., Ohguro N., Tojo K. (2009). Biol. Pharm. Bull..

[cit118] Ghasemiyeh P., Mohammadi-Samani S. (2018). Res. Pharm. Sci..

[cit119] Makoni P. A., Kasongo K. W., Walker R. B. (2019). Pharmaceutics.

[cit120] Souto E. B., Mehnert W., Müller R. H. (2006). J. Microencapsulation.

[cit121] Rani S., Rana R., Saraogi G. K., Kumar V., Gupta U. (2019). AAPS PharmSciTech.

[cit122] Vyas V., Ashby C. R., Olgun N. S., Sundaram S., Salami O., Munnangi S., Pekson R., Mahajan P., Reznik S. E. (2015). Am. J. Pathol..

[cit123] Begines B., Ortiz T., Pérez-Aranda M., Martínez G., Merinero M., Argüelles-Arias F., Alcudia A. (2020). Nanomaterials.

[cit124] Prajapati S. K., Jain A., Jain A., Jain S. (2019). Eur. Polym. J..

[cit125] Alkholief M., Kalam M. A., Anwer M. K., Alshamsan A. (2022). Pharmaceutics.

[cit126] Muljajew I., Chi M., Vollrath A., Weber C., Beringer-Siemers B., Stumpf S., Hoeppener S., Sierka M., Schubert U. S. (2021). Eur. Polym. J..

[cit127] Cheng C. J., Tietjen G. T., Saucier-Sawyer J. K., Saltzman W. M. (2015). Nat. Rev. Drug Discovery.

[cit128] Rabinow B. E. (2004). Nat. Rev. Drug Discovery.

[cit129] Medarević D., Djuriš J., Ibrić S., Mitrić M., Kachrimanis K. (2018). Int. J. Pharm..

[cit130] Tian Y., Wang S., Yu Y., Sun W., Fan R., Shi J., Gu W., Wang Z., Zhang H., Zheng A. (2022). Int. J. Pharm..

[cit131] Malamatari M., Taylor K. M. G., Malamataris S., Douroumis D., Kachrimanis K. (2018). Drug Discovery Today.

[cit132] McDonald T. O., Tatham L. M., Southworth F. Y., Giardiello M., Martin P., Liptrott N. J., Owen A., Rannard S. P. (2013). J. Mater. Chem. B.

[cit133] Hobson J. J., Savage A. C., Dwyer A. B., Unsworth C., Massam J., Arshad U., Pertinez H., Box H., Tatham L., Rajoli R. K. R., Neary M., Sharp J., Valentijn A., David C., Curley P., Liptrott N. J., McDonald T. O., Owen A., Rannard S. P. (2021). Nanoscale.

[cit134] Tao J., Chow S. F., Zheng Y. (2019). Acta Pharm. Sin. B.

[cit135] Keck C. M., Müller R. H. (2006). Eur. J. Pharm. Biopharm..

[cit136] Zhang H., Wang D., Butler R., Campbell N. L., Long J., Tan B., Duncalf D. J., Foster A. J., Hopkinson A., Taylor D., Angus D., Cooper A. I., Rannard S. P. (2008). Nat. Nanotechnol..

[cit137] McDonald T. O., Giardiello M., Martin P., Siccardi M., Liptrott N. J., Smith D., Roberts P., Curley P., Schipani A., Khoo S. H., Long J., Foster A. J., Rannard S. P., Owen A. (2014). Adv. Healthcare Mater..

[cit138] Giardiello M., Liptrott N. J., McDonald T. O., Moss D., Siccardi M., Martin P., Smith D., Gurjar R., Rannard S. P., Owen A. (2016). Nat. Commun..

[cit139] Elbaz N. M., Tatham L. M., Owen A., Rannard S., McDonald T. O. (2021). Food Hydrocolloids.

[cit140] Hoang T., Zierden H., Date A., Ortiz J., Gumber S., Anders N., He P., Segars J., Hanes J., Mahendroo M., Ensign L. M. (2019). J. Controlled Release.

[cit141] Zierden H. C., Ortiz J. I., DeLong K., Yu J., Li G., Dimitrion P., Bensouda S., Laney V., Bailey A., Anders N. M., Scardina M., Mahendroo M., Mesiano S., Burd I., Wagner G., Hanes J., Ensign L. M. (2021). Sci. Transl. Med..

[cit142] Styliari I. D., Taresco V., Theophilus A., Alexander C., Garnett M., Laughton C. (2020). RSC Adv..

[cit143] S. P. R. & Lee A. O., Tatham M., Savage A. C., Dwyer A., Siccardi M., Scott T., Manoli Vourvahis, in Conference on Retroviruses and Opportunistic Infections (CROI) - Poster 482, Boston, USA, 2018

[cit144] Elbaz N. M., Tatham L. M., Owen A., Rannard S., McDonald T. O. (2021). Food Hydrocolloids.

[cit145] Fan Y., Cui Y., Hao W., Chen M., Liu Q., Wang Y., Yang M., Li Z., Gong W., Song S., Yang Y., Gao C. (2021). Bioact. Mater..

[cit146] Barenholz Y. (2012). J. Controlled Release.

[cit147] Anselmo A. C., Mitragotri S. (2016). Angew. Chem., Int. Ed..

[cit148] Anselmo A. C., Mitragotri S. (2019). Angew. Chem., Int. Ed..

[cit149] Pantze S. F., Parmentier J., Hofhaus G., Fricker G. (2014). Eur. J. Lipid Sci. Technol..

[cit150] Feczkó T. (2021). J. Drug Delivery Sci. Technol..

[cit151] Nance E. A., Woodworth G. F., Sailor K. A., Shih T. Y., Xu Q., Swaminathan G., Xiang D., Eberhart C., Hanes J. (2012). Sci. Transl. Med..

[cit152] Elias D. R., Poloukhtine A., Popik V., Tsourkas A. (2013). Nanomed.: Nanotechnol. Biol. Med..

[cit153] Pereira Gomes C., Leiro V., Ferreira Lopes C. D., Spencer A. P., Pêgo A. P. (2018). Acta Biomater..

[cit154] Wang Y. Y., Lai S. K., Suk J. S., Pace A., Cone R., Hanes J. (2008). Angew. Chem., Int. Ed..

[cit155] Summ O., Evers S. (2013). Curr. Pain Headache Rep..

[cit156] Soundararajan A., Bao A., Phillips W. T., Perez R., Goins B. A. (2009). Nucl. Med. Biol..

